# Historic and modern genomes unveil a domestic introgression gradient in a wild red junglefowl population

**DOI:** 10.1111/eva.13023

**Published:** 2020-06-04

**Authors:** Meng Yue Wu, Gabriel Weijie Low, Giovanni Forcina, Hein van Grouw, Benjamin P. Y‐H. Lee, Rachel Rui Ying Oh, Frank E. Rheindt

**Affiliations:** ^1^ Department of Biological Sciences National University of Singapore Singapore Singapore; ^2^ School of Biological Sciences Monash University Clayton Victoria Australia; ^3^ CIBIO/InBIO Centro de Investigação em Biodiversidade e Recursos Genéticos Universidade do Porto Vairão Portugal; ^4^ Bird Group Department of Life Sciences Natural History Museum Herts UK; ^5^ Wildlife Management Research Wildlife Management Division National Parks Board Singapore Singapore; ^6^ Centre of Urban Greenery and Ecology National Parks Board Singapore Singapore; ^7^ School of Biological Sciences Centre for Biodiversity and Conservation Sciences University of Queensland Brisbane Queensland Australia

**Keywords:** admixture, conservation genomics, genetic swamping, phenotypic introgression, South‐East Asia

## Abstract

The red junglefowl *Gallus gallus* is the ancestor of the domestic chicken and arguably the most important bird species on Earth. Continual gene flow between domestic and wild populations has compromised its gene pool, especially since the last century when human encroachment and habitat loss would have led to increased contact opportunities. We present the first combined genomic and morphological admixture assessment of a native population of red junglefowl, sampled from recolonized parts of its former range in Singapore, partly using whole genomes resequenced from dozens of individuals. Crucially, this population was genomically anchored to museum samples from adjacent Peninsular Malaysia collected ~110–150 years ago to infer the magnitude of modern domestic introgression across individuals. We detected a strong feral–wild genomic continuum with varying levels of domestic introgression in different subpopulations across Singapore. Using a trait scoring scheme, we determined morphological thresholds that can be used by conservation managers to successfully identify individuals with low levels of domestic introgression, and selected traits that were particularly useful for predicting domesticity in genomic profiles. Our study underscores the utility of combined genomic and morphological approaches in population management and suggests a way forward to safeguard the allelic integrity of wild red junglefowl in perpetuity.

## 1 INTRODUCTION

The process of animal domestication has traditionally been perceived as being directed by humans, involving strong bottlenecks in domestic populations and reproductive isolation between wild and domestic forms (Driscoll, Macdonald, & O'Brien, 2009; O'Connor, 2007). Yet, a growing body of empirical and theoretical studies from both modern and archeological disciplines (e.g., Dobney & Larson, 2006) has shown that domestication of animals often involves a long‐term, ubiquitous process with no strong reproductive isolation, as exemplified by modern‐day cases of introgression between domesticated animals and their wild conspecifics such as wolves *Canis lupus* (Anderson et al., 2009; Hindrikson, Männil, Ozolins, Krzywinski, & Saarma, 2012; Randi, 2008; Randi & Lucchini, 2002; Stephens, Wilton, Fleming, & Berry, 2015; Verardi, Lucchini, & Randi, 2006), wild cats *Felis sylvestris* (Daniels, Balharry, Hirst, Kitchener, & Aspinall, 1998; Lecis et al., 2006; Randi, 2008), wild boar *Sus scrofa* (Iacolina et al., 2018), American minks *Neovison vison* (Kidd, Bowman, Lesbarreres, & Schulte‐Hostedde, 2009), and red junglefowl *Gallus gallus* (Brisbin & Peterson, 2007).

Cross‐breeding with free‐ranging domestic or feral individuals has therefore become one of the main conservation threats for the wild populations of some species (Oliveira, Godinho, Randi, & Alves, 2008), including in scenarios where declining native species recolonize parts of their former range. Gene flow from domestics or their feralized descendants into wild individuals can challenge the genomic profiles of “pure” wild populations on a local, regional, and even global scale (Allendorf, Leary, Spruell, & Wenburg, 2001; Lawal et al., 2018; Rhymer & Simberloff, 1996). The genomic makeup in domestics and wild‐type individuals is known to be different (Muir et al., 2008; Nguyen‐Phuc, Fulton, & Berres, 2016), and gene flow between the two could lead to homogenization and loss of genetic diversity between them (Gering, Incorvaia, Henriksen, Wright, & Getty, 2019; Qanbari et al., 2019). As a result, the ability to differentiate between domestic‐introgressed individuals and their nonadmixed counterparts can be of crucial importance to conservation management.

The red junglefowl *Gallus gallus* (Linnaeus, 1758) has long been considered the primary ancestor of domestic chickens (Darwin, 1875; Delacour, 1951). Molecular and archeological evidence has corroborated this hypothesis (Akishinonomiya et al., 1994, 1996; Hillel et al., 2003; Nishibori, Shimogiri, Hayashi, & Yasue, 2005; Osman & Nishibori, 2014; West & Zhou, 1988). Both the origin and date of chicken domestication are controversial topics in the literature, with the latest consensus positing that red junglefowl domestication likely occurred in Indochina (Miao et al., 2013; West & Zhou, 1988) around 4,000–10,000 years ago (Lawler, 2012; Peters, Lebrasseur, Deng, & Larson, 2016; Xiang et al., 2014). The species’ long domestication history in close proximity to naturally occurring populations has undoubtedly allowed numerous opportunities for gene flow between escaped or feral domestic chickens and wild populations of red junglefowl, which are known to readily admix (Berthouly et al., 2009; Brisbin, 1996; Brisbin & Peterson, 2007; Brisbin, Peterson, Okimoto, & Amato, 2002; Callaway, 2016; Condon, 2012; Desta, 2019; Gering, Johnsson, Willis, Getty, & Wright, 2015; Nishida et al., 2000; Peterson & Brisbin, 1998; Thakur et al., 2018).

Although the natural range of red junglefowl has considerably shrunk as a consequence of habitat fragmentation across Asia (e.g., Verma et al., 2020), they remain generally widespread and have recently recolonized areas of former occurrence (Yong, 2012). Nevertheless, their conservation status of “Least Concern” bestowed by the International Union for Conservation of Nature (IUCN)’s Red List of Threatened Species (BirdLife International, 2019) fails to account for the threat of genetic swamping from domesticated individuals and hybrid backcrosses (Brisbin & Peterson, 2007). Introgression from domestic chickens into wild red junglefowl can accumulate over time, resulting in extensive infiltration of domestic alleles into the wild gene pool. Given the ubiquitous nature of gene flow over time and domestic chickens’ abundance throughout the red junglefowl's remaining range, it has even been suggested that present‐day wild red junglefowl populations may have all been affected by domestic introgression to a certain extent (Lawler, 2012; Peterson & Brisbin, 1998).

Many previous studies have relied on morphological characters to differentiate wild red junglefowl from domestic chickens (Brisbin & Peterson, 2007; Callaway, 2016; Condon, 2012; Fernandes, Han, & Sathyakumar, 2013; Moiseyeva, Romanov, Nikiforov, Sevastyanova, & Semyenova, 2003; Peterson & Brisbin, 1998; Pheasantry & Pradesh, 2004). These studies assume that morphological traits correlate well with genomic profiles in red junglefowl. However, little research has been carried out on the reliability of these characters as an indicator of domestic introgression. While domestic chickens are phenotypically highly variable, wild junglefowl display a consistent male and female plumage type (Eaton, van Balen, Brickle, & Rheindt, 2016). Feralized offspring have been shown to exhibit wild‐type traits, despite extensive input into the gene pool from domesticated sources (Gering et al., 2015), and phenotypically intermediate individuals have been observed in areas where red junglefowl are native (Eaton et al., 2016). Additionally, backcrossing experiments between pure lines of red junglefowl and domestic chickens have produced offspring that are behaviorally and morphologically indistinguishable from their wild parents after only four generations (Brisbin & Peterson, 2007), calling into doubt the reliability of phenotypic traits for identifying non‐admixed red junglefowl. Incongruence between morphological traits and known levels of genetic admixture have also been observed in wild versus domesticated population pairs of other animal species (Daniels et al., 1998; Randi & Lucchini, 2002), corroborating decade‐old insights that morphology alone may fail to reflect evidence of introgression (Rhymer & Simberloff, 1996). Wild red junglefowl populations that remain relatively unaffected by domestic genetic swamping may now be a rarity and of conservation concern. It is therefore imperative to evaluate the magnitude and impact of introgression between wild and domestic fowl.

In the South‐East Asian island nation of Singapore, wild red junglefowl were thought to be extirpated until their reappearance on the satellite island of Ubin in the 1970s and the main island in 1999 (Wang & Hails, 2007). The first red junglefowl to recolonize Ubin Island reportedly came from Johor, Malaysia (Wang & Hails, 2007; Yong, 2012), which is separated from Ubin by a narrow sea channel approximately 800 m in width. This creates a unique study system in which an extirpated population recolonized part of its historical range from an adjacent, native locale. The Ubin Island population has been assumed to be wild because of their morphological uniformity and behavior, although they could also have originated from a population of already‐admixed junglefowl or feralized domestic chickens in Johor. The recolonization process seems to be progressing favorably, with the free‐ranging junglefowl population in Singapore having expanded across the highly urbanized country. The number of feralized domestic chickens has followed suit, whether as a result of intentional release and introduction or by chance escape. These birds can now be found in both urban spaces and parks across the island nation, where the connectivity between these subpopulations is highly dependent on the presence of park and greenland connectors. These developments have led to a thriving present‐day population that exhibits a spectrum of morphological characters. It is therefore possible that the perceived increase in Singapore's free‐roaming red junglefowl population is in fact largely due to increases in feral or admixed individuals (Johnsson et al., 2016). Admixture between feral individuals with their wild counterparts leaves management authorities with the challenge of how to differentiate between red junglefowl of conservation importance and domestic hybrids devoid of such a perceived value.

In this study, we investigated a panel of 81 red junglefowl and chickens to evaluate the incidence of genomic admixture between feral individuals and wild junglefowl that have recolonized Singapore and to verify whether morphology is a reliable indicator of genotype in red junglefowl. Firstly, we generated millions of genome‐wide single nucleotide polymorphisms (SNPs) from whole‐genome resequencing (WGR) and double‐digest restriction site‐associated DNA sequencing (ddRADSeq) spanning 79 red junglefowl and chickens across Singapore and identified subsets of markers that reflect the domestic–wild genomic continuum. Then, we used “ancient DNA” sequencing methods to genotype two historic museum samples (~110‐150 years old) from Peninsular Malaysia as a genomic reference for wild red junglefowl relative to our recently sampled individuals. To the best of our knowledge, this is the first time that historic museum individuals have been used as a genomic anchor for determining the true genotype of wild junglefowl, which is a substantial improvement over the conventional use of present‐day samples of unknown admixture history. While it is uncertain that these historic samples had never experienced any admixture from domesticated sources, they hail from a time when there was a much larger buffer area between wild habitats and human settlements. Therefore, they serve as suitable references to detect modern excess introgression that would have occurred during the Anthropocene and led to more significant domestic admixture in wild populations. We then identified discrete morphological traits that can be easily evaluated visually to correlate morphology with genomic profiles across our dataset and identify the most informative traits.

## 2 MATERIALS AND METHODS

### 2.1 Sample collection

**FIGURE 1:**
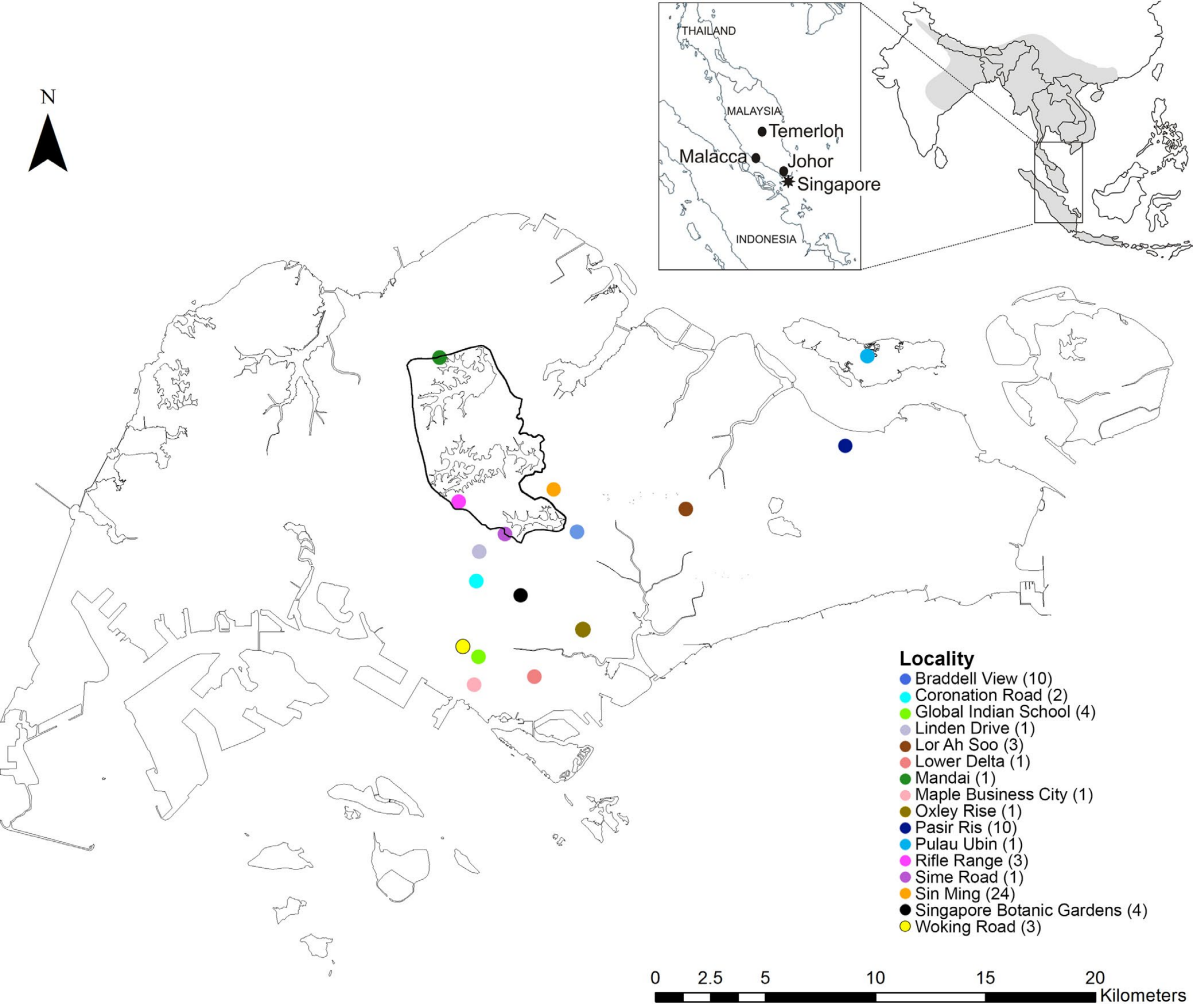
Distribution map of free‐roaming red junglefowl (*Gallus gallus*) sampled across Singapore. The number of individuals from each location is indicated in brackets. Central Catchment area is outlined in black. The native range of the red junglefowl is colored gray in the upper right insert

We analyzed a total of 81 samples in our study, including 79 contemporary and two historic DNA samples. Specifically, we collected blood from 70 free‐roaming birds (61 adults, 9 juveniles) encompassing the whole spectrum of morphology from domestic to wild type observed in Singaporean red junglefowl populations (Figure 1). The birds were caught with leg‐hold traps and sampled within ten minutes before being released. In Singapore, the Wild Animal and Birds Act (2000) together with the Animal and Birds Act (2007) restrict the number of domestic chickens through the nationwide ban of rearing on most properties and through occasional removal. Given that the entire country is urbanized, with no more rural settlements, Singapore lacks any semiferal “village chicken” populations. Thus, we obtained nine domestic chickens from an indoor poultry farm in Singapore, comprising two individuals from a proprietary line of Silkies as well as two and five individuals from commercial lines of Bovan Browns and Bovan Whites, respectively (Hendrix Genetics, Netherlands). We further sampled dried toepads from two red junglefowl collected between 1870 and 1911 from Peninsular Malaysia and preserved at the Natural History Museum at Tring (UK) and Lee Kong Chian Natural History Museum (Singapore; Table S1). All sampling protocols were in accordance with institutional ethics regulations and guidelines.

### 2.2 Morphological scoring

**FIGURE 2:**
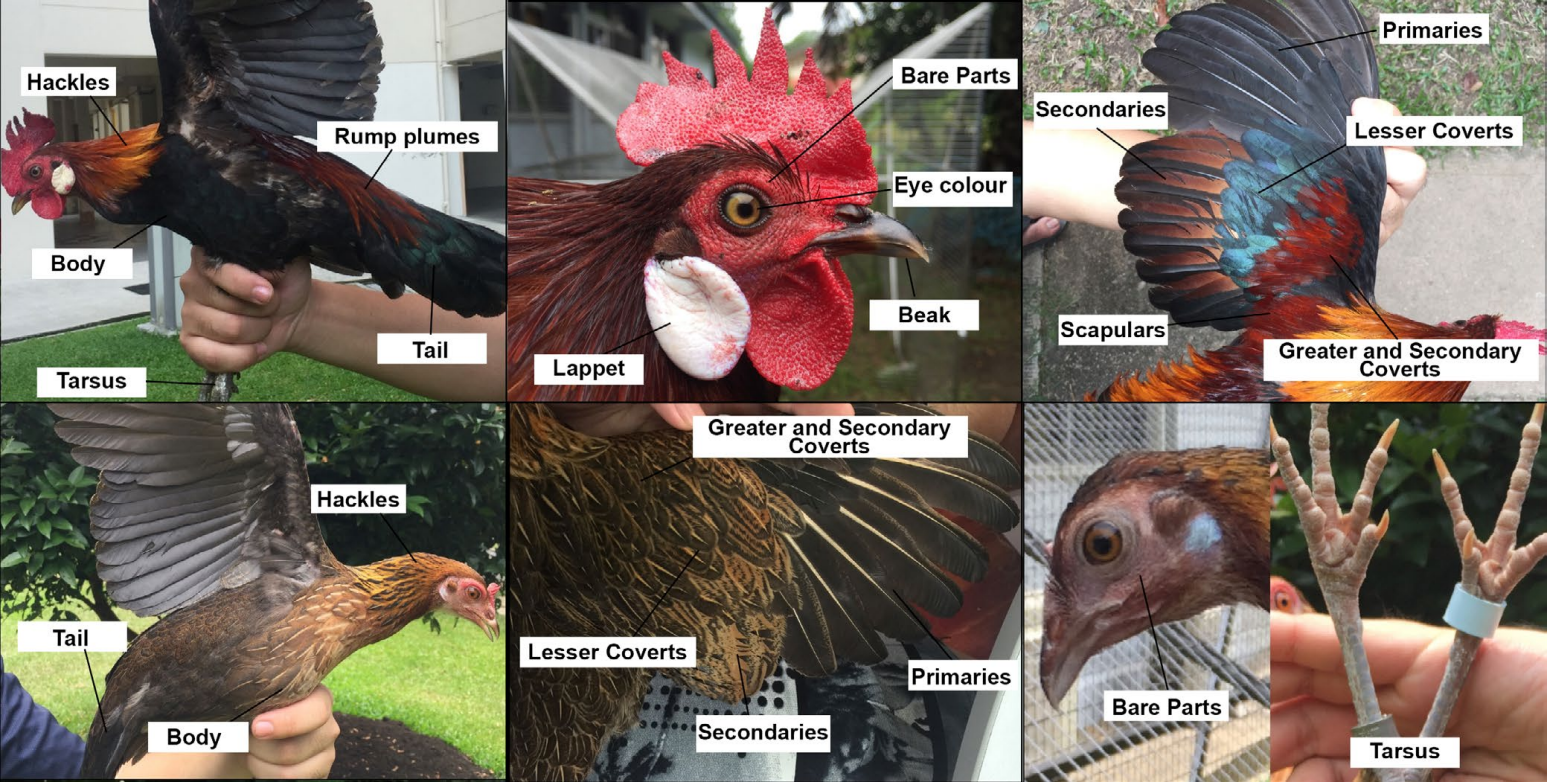
Illustration of body parts scored morphologically (see Table 1) in sampled individuals of *Gallus gallus* (male above; female below). All photographs exhibit “wild” character states, with the exception of the trait “secondaries,” for which not a single male red junglefowl showcased a “wild” state

We scored a total of 61 wild‐caught red junglefowl individuals and the nine domestic chickens from photographs taken at the time of capture. We scored only adults as juveniles and chicks exhibit large variation in their plumage. Adults with any scored trait obscured in photographs were also removed. We did not score museum samples morphologically because of observed foxing and bleaching of colors in bare parts and plumage, although we did ascertain that the two museum samples were characterized by plumage traits typical of wild red junglefowl. We employed categorical morphological traits with the intention to increase their utility to field practitioners and management agencies. All adults were scored on the basis of distinguishing traits identified from the existing literature (Brisbin & Peterson, 2007; Eaton et al., 2016; Glenister, 1971; Nishida et al., 2000; Robson, 2008; Wells, 1999). Different traits were used for females and males due to the species’ strong sexual dimorphism (Figure 2, Table 1). The scores were then normalized in order to compare the two sexes, where individuals with an appearance closer to the wild type were assigned a higher score along a continuum from 0 to 100. Overall, morphological scores were expressed as a fraction of the total possible score to enable cross‐comparison between sexes.

**TABLE 1:** Morphological traits scored for males and females (see also Figure 2).

Morphological trait		Males			Females		
		Wild	Domestic	Scoring	Wild	Domestic	Scoring
Color	Hackles (Hack)	Golden‐yellow in a gradient	Other colors or pattern	0, 1	Blackish and golden buff	Other colors or pattern	0, 1
	Body (Body)	Dark green	Other colors; 0.5 is awarded if ~ 50% have the typical color	0, 0.5, 1	Brown with blackish vermiculations	Other colors; 0.5 is awarded if the color is a shade darker or lighter than in wild	0, 0.5, 1
	Tail (Tail)	Dark green	Other colors; 0.5 is awarded if ~50% have the typical color	0, 0.5, 1	Dark brown	Other colors	0, 1
	Bare Parts (BP)	Red	Other colors	0, 1	Pink	Other colors; 0.5 is awarded if ~50% have the typical color	0, 0.5, 1
	Tarsus (TarC)	Gray	Other colors	0, 1	Gray	Other colors	0, 1
	Primaries (Pri)	Black	Other colors	0, 1	Black	Other colors	0, 1
	Secondaries (Sec)	Orange	Other colors; 0.5 is awarded if ~50% have the typical color	0, 0.5, 1	Brown with blackish vermiculations	Other colors	0, 1
	Lesser Coverts (LC)	Green	Other colors	0, 1	Brown with blackish vermiculations	Other colors	0, 1
	Greater and Secondary Coverts (GSC)	Maroon	Other colors	0, 1		Other colors	0, 1
	Scapulars (S)	Maroon	Other colors; 0.5 is awarded if ~50% have the typical color	0, 0.5, 1	–	–	–
	Rump Plumes (RP)	Orange Red	Other colors	0, 1	–	–	–
	Eye Color (Eye)	Hazel	Other colors	0, 1	–	–	–
	Lappet (Lap)	White	Other colors; 0.5 is awarded if ~50% have the typical color	0, 0.5, 1	–	–	–
	Beak (Beak)	Horn	Other colors; 0.5 is awarded if ~50% have the typical color	0, 0.5, 1	–	–	–
Other	Presence of Tarsal Spur (TS)	Elongated	Short or absent	0, 1	–	–	–
	White Rump Feathers (WRF)	Present	Absent	0, 1	–	–	–
	Presence of Wattles and Comb (WC)	–	–	–	Absent	Present; 0.5 is awarded if either one occurs	0, 0.5, 1

### 2.3 DNA extraction, library preparation, and sequencing

This study combines three different types of sequencing techniques: WGR of historic museum samples (*n* = 2), WGR of contemporary samples (*n* = 58), and ddRADSeq of contemporary samples (*n* = 21). Each data type was generated using a specific protocol detailed below.

We extracted genomic DNA from the 79 contemporary blood samples and two museum toepad specimens using the DNeasy® Blood & Tissue Kit (Qiagen) following the manufacturer's protocol and further applied an additional RNase treatment. Extractions from museum toepads were carried out with further in‐house modifications (Chattopadhyay, Garg, Mendenhall, & Rheindt, 2019; Chattopadhyay, Garg, Soo, et al., 2019). The amount of ATL buffer, proteinase K, and AL buffer was doubled, and incubation temperature was increased to 65°C to aid in the digestion of the toepad clippings. Incubation at −20°C after the addition of 100% molecular ethanol helped maximize DNA retrieval. DNA was eluted in two sets of 50 ul molecular water, making the final volume 100 ul.

WGR library preparation for a subset of 58 contemporary blood samples was carried out by using either the NEBNext® Ultra™ DNA Library Prep Kit for Illumina® (New England Biolabs) at the A*STAR Genome Institute of Singapore or the NEBNext® Ultra™ II FS DNA Library Prep Kit for Illumina® (New England Biolabs) in‐house at the National University of Singapore, selecting for a 200–350 bp insert size. We chose samples for WGR with a view to achieving a comprehensive geographic coverage across Singapore. We additionally created ddRADSeq libraries (Peterson, Weber, Kay, Fisher, & Hoekstra, 2012) for the remaining 21 contemporary blood samples following Low et al. (2018).

For library preparation of the two museum toepad samples, we first carried out an FFPE DNA repair reaction using the NEBNext® FFPE DNA Repair Mix (New England Biolabs). A cleanup was carried out by adding AMPure XP beads to the FFPE DNA repair reaction mix, and DNA was eluted in 55.5 ul. The volumes of End Repair Reaction Buffer and End Prep Enzyme Mix were increased to 6.5 and 3.0 ul, respectively, and added to the FFPE repaired DNA from the NEBNext® Ultra™ DNA Library Prep Kit for Illumina® (New England Biolabs). Size selection was not carried out after adaptor ligation. PCR and following cleanup were conducted according to the manufacturer's protocol, but with DNA being eluted in 17 ul of nuclease‐free water. We also prepared negative controls for both extraction and library preparation to ensure no modern DNA contaminates the endogenous DNA of the museum toepads.

Concentrations were quantified using either a Qubit® 2.0 High Sensitivity DNA Assay or Qubit® 2.0 Broad Range DNA Assay (Invitrogen), and library fragment size distributions were checked using a Fragment Analyzer (Advanced Analytical Technologies). Once checked, libraries were sequenced at the A*STAR Genome Institute Singapore or NovogeneAIT Genomics (Singapore) on the Illumina HiSeq 4000 platform to produce 150‐bp paired‐end reads.

### 2.4 Quality filtering and SNP calling

Following initial quality assessment of raw sequence data using *FastQC* (Babraham Bioinformatics, USA), we retained all sequences without truncation. The final quality‐filtered dataset consisted of more than three billion reads with an average of 87 million reads per individual for WGR and seven million reads per individual for ddRADSeq sets. Reads were 150 bp long, with the exception of museum samples that had an average read length of 92 bp. WGR recovered an average sequencing coverage of 10×.

For the 21 individuals subjected to ddRADSeq, we first demultiplexed and filtered raw reads using the *process_radtags* command as implemented in Stacks v1.4 (Catchen, Hohenlohe, Bassham, Amores, & Cresko, 2013). Across all 81 samples, we removed adaptor sequences with cutadapt (Martin, 2011) and aligned both ddRADSeq and WGR reads to Gallus_gallus‐5.0 (GenBank accession: GCA_000002315.3) (International Chicken Genome Sequencing Consortium, 2004) using BWA‐MEM v0.7.17 (Li, 2013). Low‐quality reads (MAPQ score < 20) were filtered with SAMtools v1.6‐1 (Li et al., 2009) to ensure unique mapping. Picard v2.17.3 (http://broadinstitute.github.io/picard/) was subsequently used to assign read group information and mark duplicates. Lastly, we used RealignedTargetCreator and IndelRealigner as implemented in the Genome Analysis Toolkit (GATK) v3.8‐0 (Broad Institute, USA) (McKenna et al., 2010) to realign and refine the original alignment. The output bam format files were checked in Qualimap v2.2.1 (Okonechnikov, Conesa, & García‐Alcalde, 2015) for mapping quality and sequencing bias before variant calling.

Museum samples are known to experience severe *post mortem* DNA damage that can confound downstream analysis necessitating the application of sophisticated tools (reviewed in Billerman & Walsh, 2019; MacHugh, Larson, & Orlando, 2017; Pääbo et al., 2004). In order to minimize these artifacts, we rescaled the quality scores of these samples using a Bayesian statistical model of DNA damage as implemented in mapDamage 2.0 (Jónsson, Ginolhac, Schubert, Johnson, & Orlando, 2013).

There are three different types of data used in this study: WGR from historic museum samples, WGR from contemporary samples, and thousands of genome‐wide SNPs harvested from contemporary samples through ddRADSeq. As data from both museum samples and ddRADSeq were more sparsely distributed across the genome, attempts to combine them and retain only overlapping loci resulted in a large loss of informative sites. Therefore, we produced three datasets: (1) a first dataset only comprising WGR samples (contemporary and historic; *n* = 60) to minimize data loss while keeping the museum samples as a reference for the genomic profile of wild red junglefowl from >100 years ago; (2) a second dataset comprising only contemporary WGR samples (*n* = 58) to call SNPs using stricter filters that cannot viably be applied to highly fragmented historic museum sequences; (3) and a third dataset comprising all contemporary samples (WGR and ddRADSeq; *n* = 79) to test whether the addition of samples at lower locus counts yields concurring results.

ANGSD v0.923 is designed to conduct population genetic analysis for low coverage data, suitable for the nature of our historic samples (Billerman & Walsh, 2019; Korneliussen, Albrechtsen, & Nielsen, 2014). We used ANGSD to call SNPs with different filters applied for each dataset: (1) The first filter regime (90% presence across individuals, MinDepth 3, MinMapQ 30, MinQ 30, Minor Allele Frequency 0.01, geno_depth 3) was designed to be lenient to allow SNP capture from degraded museum samples; (2) the second filter regime was stricter (95% presence across individuals, MinDepth 3, MinMapQ 30, MinQ 30, Minor Allele Frequency 0.05, geno_depth 3) to be applied to contemporary DNA sample sets only in order to verify results found in (1); (3) and the third filter regime was a modification of the second, designed to test whether the same results can be obtained with an increased sample size and reduced genome representation (90% presence across individuals, MinDepth 3, MinMapQ 30, MinQ 30, Minor Allele Frequency 0.05, geno_depth 3).

We removed single members of SNP pairs with a pairwise linkage disequilibrium correlation coefficient higher than 0.5 as measured in PLINK v1.90 using a window size of 25 and a step size of 10 (Chang et al., 2015; Purcell et al., 2007). Individuals with high missing data (>15%) as determined in PLINK were also removed, leading to the exclusion of six modern individuals from further analysis. Finally, we estimated pairwise kinship coefficients using maximum likelihood estimation (Choi, Wijsman, & Weir, 2009; Milligan, 2003) in the SNPRelate R package (Zheng et al., 2012) to prevent false‐positive associations (Choi et al., 2009). Kinship analysis found that 18 individual pairs were siblings or parent–offspring. Close kin were removed during exploratory analysis, and the results were compared to datasets without kin removal. As no significant differences were found in observed trends between the two datasets (including and excluding close kin; Figures S1 and S2), close kin were retained in all downstream analyses.

After quality filtering, 55, 52, and 74 individuals were retained in datasets 1, 2, and 3, respectively. Oversampling from both Sin Ming and Pasir Ris caused principal component analysis (PCA) of all datasets to show strong localization effects during preliminary inspection (Figure S3). Individuals from these two sites were therefore reduced to three each to account for oversampling bias, with selection based on the availability of morphology scores and their placement in the PCA plots to be representative of local variability. The two museum samples passed quality filtering, with most inserts being shorter than 150 bp long, creating substantial overlap between paired‐end sequences that helped reduce sequencing errors (Besnard et al., 2016). All downstream analyses were carried out on these reduced datasets. Therefore, in total, 35, 33, and 48 individuals were used in this study for dataset 1 (9,900,037 SNPs), dataset 2 (4,441,673 SNPs), and dataset 3 (7,812 SNPs), respectively.

### 2.5 Population genomic approaches

We assessed population subdivision in Singaporean free‐roaming junglefowl by running PCA in SNPRelate (Zheng et al., 2012) for each of the datasets. For comparison, we also included an additional 98 WGR samples of red junglefowl and various chicken breeds available on GenBank using the variant calling filters of 70% presence across individuals, MinDepth 3, MinMapQ 30, MinQ 30, Minor Allele Frequency 0.01, and geno_depth 3 (Table S2).

We additionally conducted Bayesian clustering in STRUCTURE (Pritchard, Stephens, & Donnelly, 2000) with a random subset of 100,000 SNPs for each dataset, employing the wrapper Structure_threader v1.2.4 (Pina‐Martins, Silva, Fino, & Paulo, 2017) to parallelize all runs. Clustering analysis was run for *K* = 1–5 with 10 replicates for each *K*, employing a burn‐in of 100,000 generations and 500,000 further Monte Carlo Markov chain (MCMC) generations. To aggregate replicates for each *K*, we ran STRUCTURE output through CLUMPAK (Kopelman, Mayzel, Jakobsson, Rosenberg, & Mayrose, 2015) using the FullSearch algorithm for *K* = 1–3 and Greedy algorithm for *K* = 4 and 5. We did not run tests for optimal *K* values (e.g., Evanno, Regnaut, & Goudet, 2005) as it was not our purview to investigate population genetic structure in Singaporean *Gallus* populations; instead, we focused on the genomic component that best reflects domestic admixture.

### 2.6 Relating morphology to genomics

Eigenvalues from the principal component showing the wild–domestic continuum in the genomic PCA were extracted and used as a proxy for relating morphology to genotype. Linear regression was used to test whether overall morphology scores corresponded to these eigenvalues.

We also constructed linear regression models to identify morphological traits that best predicted the amount of domestic genomic admixture of red junglefowl. A large number of traits were scored (males = 16 traits; females = 10 traits), but dataset 1 only comprised 16 adult males and 12 adult females; thus, we defined a subset of morphological traits to efficiently make use of the available degrees of freedom. For this purpose, we first ran a PCA on morphological data, using the *FactoMineR* and *factoextra* packages (Kassambara & Mundt, 2017; Le, Josse, & Husson, 2008). Traits that exhibited an above‐average contribution to the principal component showing the domestic–wild continuum in the morphological PCA were retained for regression modeling with genomic data. We generated regression models in which eigenvalues from the genomic continuum of dataset 1 were regressed against different combinations of the identified morphological traits (Table S3). Tarsus color (TarC) in females did not initially emerge as an above‐average contributor to morphological PCA, but during regression modeling of single traits, it was found to have a lower Akaike's information criterion (AIC) value than multiple traits identified from the PCA as large contributors in the domestic–wild continuum (Table S3). Therefore, we decided to include female tarsus color in our modeling given its presumed importance as an introgressed trait from gray junglefowl *Gallus sonneratii* (Eriksson et al., 2008). All models fitted are presented in Table S3. All statistical analyses and modeling were carried out in R version 3.3.2 (R Core & Team, 2019).

## 3 RESULTS

### 3.1 Population genetic structure of Singaporean free‐roaming junglefowl

**FIGURE 3:**
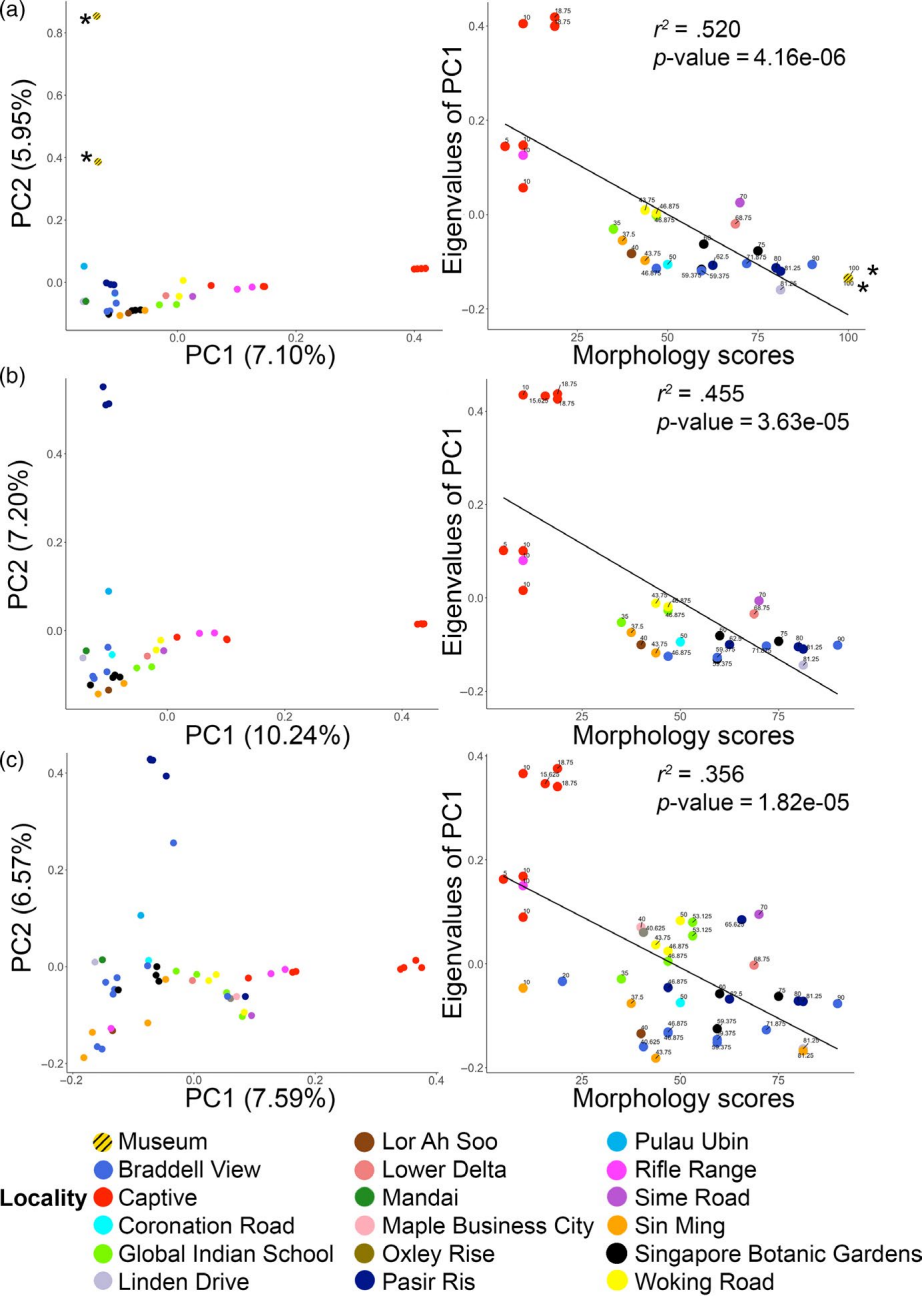
Principal component analysis of the genomic dataset (left) and linear regression of morphology scores against eigenvalues extracted from PC1 (right): (a) whole‐genome resequenced individuals including museum samples (*n* = 35) based on 9,900,037 single nucleotide polymorphisms; (b) whole‐genome resequenced individuals excluding museum samples (*n* = 33) based on 4,441,673 single nucleotide polymorphisms; (c) modern individuals excluding museum samples but including individuals from restriction‐associated DNA sequencing (*n* = 48) based on 7,812 single nucleotide polymorphisms. The percentage of total variation explained by each principal component is shown in brackets. Coefficients of determination and *p*‐values are reported for each linear regression plot. Museum samples are labeled with an asterisk (*)

We observed a genomic continuum along the first principal component (PC1) (Figure 3) from free‐roaming individuals, including those represented by museum samples, to domestic captive‐bred individuals. When plotted among a larger global body of GenBank samples comprising red junglefowl and domestic chickens from diverse backgrounds, Singaporean free‐roaming red junglefowl mostly clustered separately from the rest of the global population, possibly because of inequalities of sample size (Figure S4). Nevertheless, they consistently clustered closest to other red junglefowl or chickens from the Asian region (Figure S4). Singaporean captive layer individuals were found to be similar to layer and white leghorn individuals from other studies, reflecting their commercial lineage.

**FIGURE 4:**
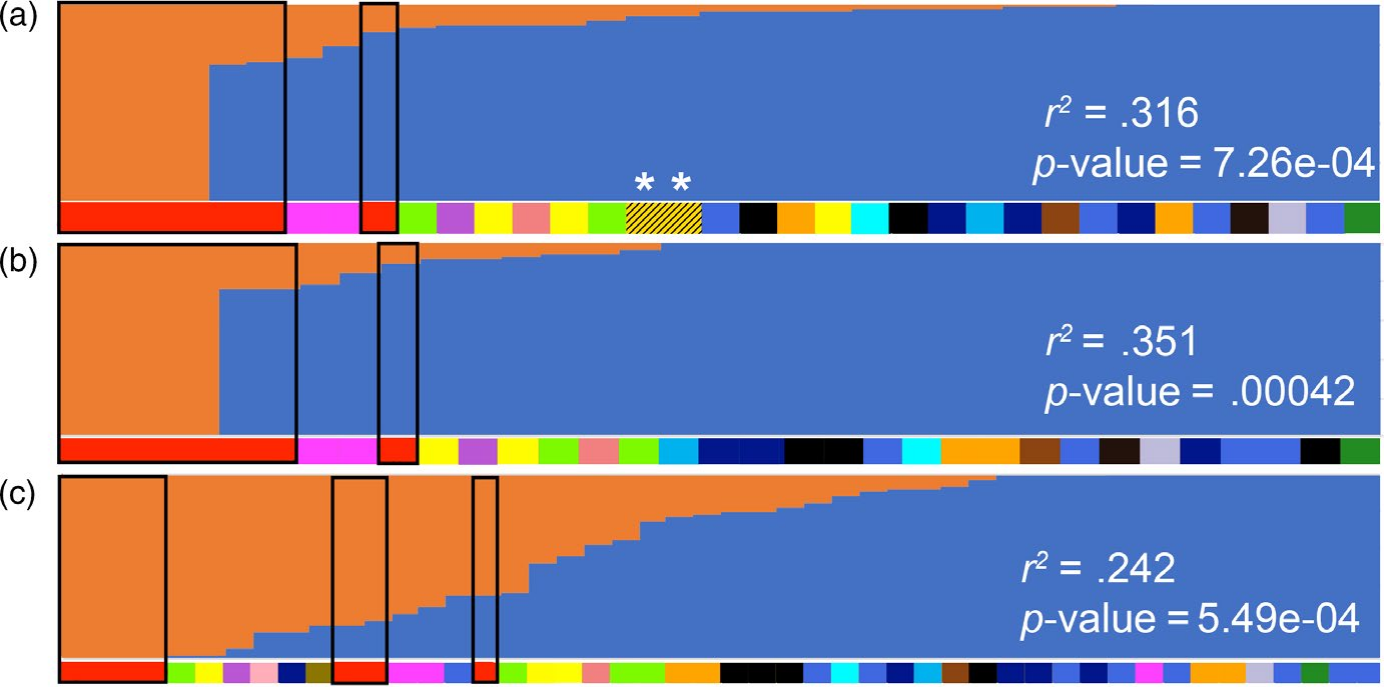
STRUCTURE plot at *K* = 2: (a) whole‐genome resequenced individuals including museum samples (i.e., dataset 1; *n* = 35) based on 100,000 single nucleotide polymorphisms; (b) whole‐genome resequenced individuals without museum samples (i.e., dataset 2; *n* = 33) based on 100,000 single nucleotide polymorphisms; (c) whole‐genome resequenced individuals plus individuals from restriction‐associated DNA sequencing (RADSeq) without museum samples (i.e., dataset 3; *n* = 48) based on 7,812 single nucleotide polymorphisms. Coefficients of determination and *p*‐values from regression analysis of morphology scores against *q* scores are given in each STRUCTURE plot. Museum samples are labeled with an asterisk (*), and domestic samples are highlighted by black boxes. Colors refer to localities as shown in Figure 3

Bayesian analysis performed in STRUCTURE at *K* = 2 also revealed a genomic continuum in free‐roaming junglefowl, with historic samples and individuals exhibiting a wild‐type morphology on the one end and captive individuals with a domestic morphology on the other (Figure 4). The genomic continuum was much more gradual in dataset 3, probably because its underlying SNP set is not only much smaller (7,812 SNPs in dataset 3 versus >4 million SNPs in dataset 2) but is also characterized by much lower linkage disequilibrium than that of the other two datasets (Figure 4).

This genomic continuum between wild‐type and domestic individuals was not observed at higher *K* values tested (Figure S5). As we are only interested in the one population genetic component that reflects domestic admixture, we ignored divisions at higher *K* values and ranked the membership coefficient (*q*) values in STRUCTURE analysis for *K = *2 from lowest to highest (range from 0 to 1), with a higher *q‐*score being indicative of less domestic ancestry in an individual (Figure 4) and vice versa. Although the two historic museum samples from Malaysia displayed evidence consistent with some domestic introgression, they still exhibited *q* scores of ≥ 0.9, which we adopt as a threshold for assigning “wild‐type” individuals from the modern sample set for the purpose of this study. Considering this threshold, the percentage of individuals with *q* ≥ 0.9 was around 60%, 73%, and 40% for datasets 1, 2, and 3, respectively.

### 3.2 Characterizing the domestic–wild morphological and genomic continuum

A total of 62 adults out of 79 genotyped individuals were morphologically scored (Table 2). We found a significant correlation between morphological scores and genomically inferred domesticity (Figure 3), which was quantified using PC1 eigenvalues as a proxy in all three datasets.

**TABLE 2:** Morphological percentage scores for 62 adult individuals according to the criteria listed in Table 1.

Sample	Locality	Morphology score (%)	Sample	Locality	Morphology score (%)
BL01	Captive	5.00	RJF43_LAS	Lor Ah Soo	59.38
BL02	Captive	10.00	RJF44_WR	Woking Road	43.75
RJF01_GR	Sin Ming	37.50	RJF45_WR	Woking Road	46.88
RJF02_GR	Sin Ming	18.75	RJF46_BV	Braddell View	46.88
RJF04_OR	Oxley Rise	40.63	RJF47_BV	Braddell View	46.88
RJF06_MBC	Maple Business City	40.00	RJF48_BV	Braddell View	20.00
RJF07_SR	Sime Road	70.00	RJF49_DE	Singapore Botanic Garden	59.38
RJF10_SM	Sin Ming	75.00	RJF50_LD	Linden Drive	81.25
RJF11_SM	Sin Ming	30.00	RJF51_BV	Braddell View	40.63
RJF14_SM	Sin Ming	43.75	RJF54_RV	Pasir Ris	65.63
RJF15_SM	Sin Ming	20.00	RJF55_RV	Pasir Ris	62.50
RJF19_SM	Sin Ming	65.63	RJF58_BV	Braddell View	59.38
RJF20_SM	Sin Ming	62.50	RJF59_BV	Braddell View	90.00
RJF21_SM	Sin Ming	10.00	RJF61_BV	Braddell View	62.50
RJF22_SM	Sin Ming	10.00	RJF62_RV	Pasir Ris	46.88
RJF24_SM	Sin Ming	65.00	RJF63_BV	Braddell View	59.38
RJF25_SM	Sin Ming	50.00	RJF66_PP	Pasir Ris	80.00
RJF26_LP	Sin Ming	81.25	S01	Captive	10.00
RJF27_LP	Sin Ming	75.00	S02	Captive	10.00
RJF28_LP	Sin Ming	70.00	WL01	Captive	18.75
RJF29_LP	Sin Ming	55.00	WL02	Captive	18.75
RJF30_LP	Sin Ming	45.00	WL03	Captive	15.63
RJF32_SC	Sin Ming	40.00	WL04	Captive	10.00
RJF33_LAS	Lor Ah Soo	71.88	H0031_PP	Pasir Ris	81.25
RJF34_GIS	Global Indian School	53.13	H0032_PP	Pasir Ris	80.00
RJF35_GIS	Global Indian School	46.88	H0033_SBG	Singapore Botanic Garden	60.00
RJF31_CR	Coronation Road	46.875	H0034_SBG	Singapore Botanic Garden	75.00
RJF38_RR	Rifle Range	10.00	H0035_BV	Braddell View	71.88
RJF39_GIS	Global Indian School	35.00	H0036_SM	Sin Ming	78.13
RJF40_WR	Woking Road	50.00	H0037_LDel	Lower Delta	68.75
RJF42_LAS	Lor Ah Soo	40.00	H0038_CR	Coronation Road	50.00

Lower scores are representative of a domestic phenotype and higher scores of individuals with wild‐type morphological characteristics.

The regression model explained 52% of the observed variation in dataset 1 (Figure 3a). A similar continuum appeared when dividing population genetic variation into two genetic clusters in STRUCTURE (*K* = 2, Figure 4a), with corresponding *q* values closely correlating with morphology (Figure 4a). When excluding museum samples (dataset 2), we defined a set of individuals from Pasir Ris as the most representative of the “wild” genotype owing to their close clustering position with the museum samples and distance from the domestic individuals in dataset 1 along PC1 (Figure 3a). We observed a similar domestic–wild continuum along PC1 (Figure 3b), and regression analyses showed a close relationship between morphological scores and both PC1 eigenvalues and *q* values from STRUCTURE (Figures 3b and 4b). We detected the same relationship in dataset 3 when considering a larger sample size including the full ddRADSeq dataset (Figures 3c and 4c). Comparable overall trends in PCA, STRUCTURE, and regression plots emerged when removing closely related kin from each dataset (Figures S1 and S2).

We conducted the same analysis for datasets 2 and 3 after removing the captive individuals from both samples, which resulted in the emergence of the same genomic continuum along the second principal component (Figure S6). Again, we found a significant correlation between morphological scores and genomically inferred domesticity in dataset 2 but not in dataset 3 after removal of captive individuals (Figure S6).

### 3.3 Identifying morphological traits that best predict genomic domestic contribution

**FIGURE 5:**
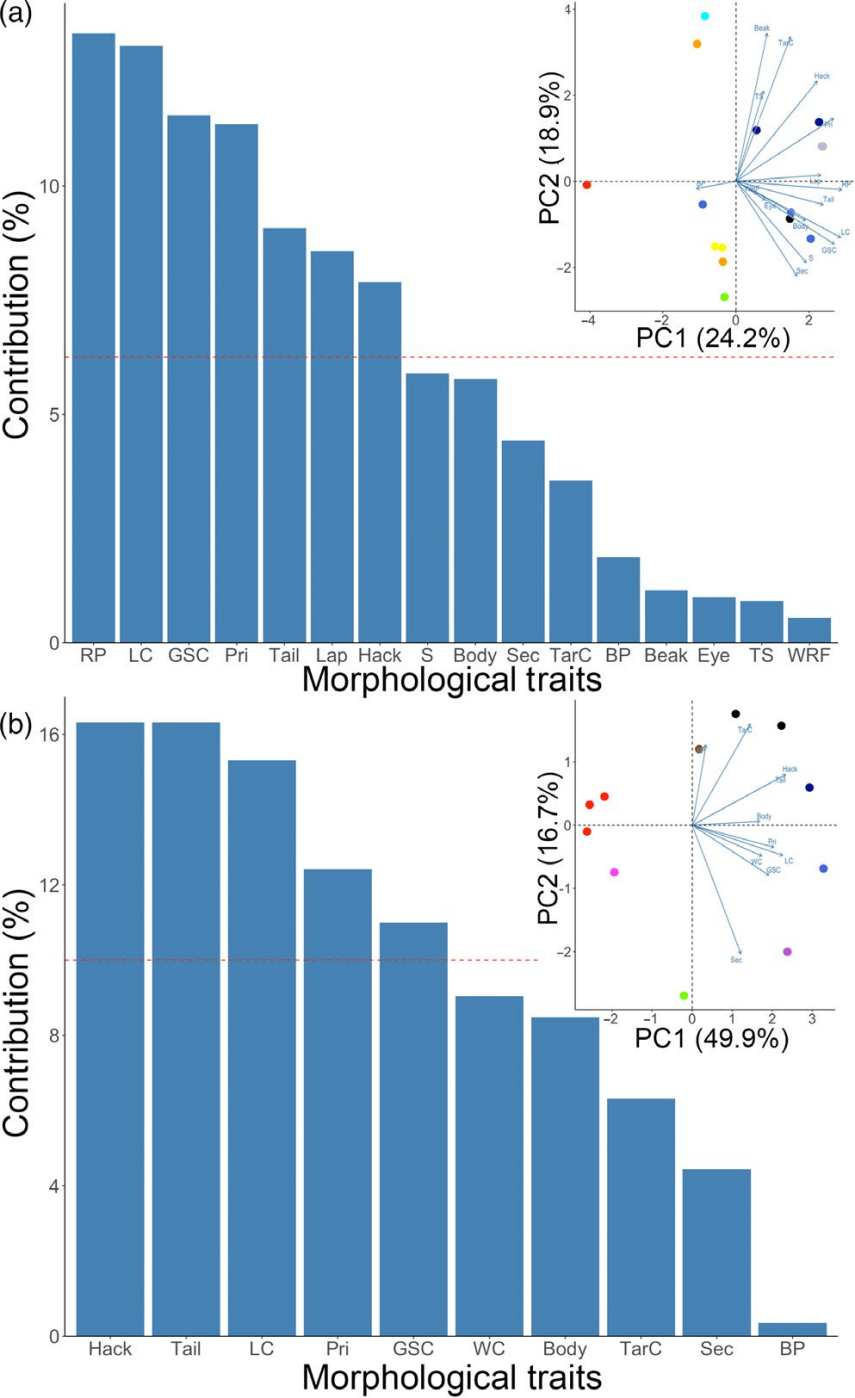
Principal component analysis biplot of all scored individuals in dataset 1 using morphological data. Contribution of each morphological trait to the variability along PC1 in males (*n* = 16) (a) and females (*n* = 12) (b). The expected average contribution is denoted by the red dashed line. The percentage of total variation explained by each principal component is shown in brackets. Abbreviations for morphological traits follow Table 1. Colors refer to localities as shown in Figure 3

Our PCA built from morphological traits exhibited a domestic–wild continuum along PC1 for both males and females similar to the continuum produced with genetic data (Figure 5). We specifically identified seven out of 16 male morphological traits that exhibited an above‐average contribution to PC1 and five out of ten female morphological traits with an above‐average contribution to PC1 (Figure 5). AIC model selection indicated that coloration of tail, primaries, and lappet best predict the genomic profile in males, whereas tarsus and primary feather coloration were found to be the best predictors of genomic profile in females (Table 3).

**TABLE 3:** Top five linear models for each sex based on Akaike's information criterion (AIC).

Model		*df*	AIC
Males	Eigenvalues ~ Tail+Pri + Lap	5	−27.900529
	Eigenvalues ~ Hack+Tail + Pri+Lap	6	−26.118934
	Eigenvalues ~ Tail+Pri + RP+Lap	6	−25.978275
	Eigenvalues ~ Tail+Pri + GSC+Lap	6	−25.956527
	Eigenvalues ~ Tail+Pri + LC+Lap	6	−25.915366
Females	Eigenvalues ~ TarC+Pri	4	−21.479253
	Eigenvalues ~ LC+Pri + TarC	5	−20.563989
	Eigenvalues ~ Pri+GSC + TarC	5	−19.811125
	Eigenvalues ~ Hack+Tail + Pri+TarC	5	−19.480188
	Eigenvalues ~ Hack+Pri + TarC	5	−19.480188

All fitted models and acronyms and abbreviations for traits can be found in Table S3.

Abbreviation: *df*, degree of freedom.

## 4 DISCUSSION

### 4.1 Historic specimens reveal domestic admixture in Singapore's free‐roaming Junglefowl

Our study provides the first genomic assessment of domestic admixture in a free‐roaming population within the native distribution of red junglefowl. The species recently recolonized Singapore after having been extirpated for many decades (Wang & Hails, 2007), but continuous domestic contributions have cast doubt on the extent of domestic admixture in Singapore's population.

Crucially, we generated a genomic reference for “wild red junglefowl” based on two historic museum samples collected in adjacent Peninsular Malaysia in the late 19th and early 20th centuries. Even though they are most likely not “pure red junglefowl” given the long domestication history across the species’ range, extensive domestic introgression in recent decades is likely to have further affected most wild populations of red junglefowl (Brisbin & Peterson, 2007; Lawler, 2012). We considered it essential to anchor our genomic analysis of junglefowl to historic samples from adjacent areas collected at a time when pristine habitats were likely separated from areas of human encroachment by more substantial buffers, reducing the potential of contact with domestic stock and thus the extent of domestic introgression (Corlett, 1992). This practice is superior to conventional approaches of using present‐day samples of unknown admixture history as wild anchors, solely on the basis of their morphological characters and collection locality.

Using this approach, we found that free‐roaming red junglefowl in Singapore are characterized by a domestic–wild genomic continuum with varying levels of introgression. We did not observe any clear spatial patterns that controlled this continuum: Individuals with a genomic signature typical of wild junglefowl were interspersed across the whole study area among others with higher domestic contributions. For example, individuals from a 1.33‐km^2^ area (Sin Ming) exhibited the entire gamut of domestic introgression levels present in Singapore (Figures 3, 4 and 3, 4; Figure S3). Nevertheless, areas where most of the genomically and morphologically wild‐type individuals were detected were often around Singapore's largest nature reserve, the Central Catchment (Figure 1).

The level of domestic introgression in Singapore's free‐roaming population appears lower as compared to a previous study on domestic admixture of a free‐ranging population on Kauai, Hawaii, with largely wild‐type morphological traits (Gering et al., 2015), although a direct comparison of Gering et al.’s (2015) data and ours was not possible. As the Hawaiian Islands are distant from the native range of junglefowl, Kauai population's domestic origin is in no doubt (Pyle & Pyle, 2009; Thomson et al., 2014), and it has been suspected to be possibly of Pacific or European descent (Gering et al., 2015). The wild‐type appearance of these fowl illustrates the capability of populations to exhibit an ancestral phenotype after generations of wild roaming (Brisbin & Peterson, 2007; Gering et al., 2015) even though their genomic signature may retain considerable levels of domestic admixture.

### 4.2 Morphology as an indicator of genomic profile in *Gallus*

The advantage of morphology‐based introgression assessments is their ease of application relative to the expensive and slower process of genomic screening. In this study, we utilized genome‐wide markers to gauge the reliability of morphological traits for inferring levels of domestic introgression into red junglefowl.

Our PCA and regression analysis clearly divided the captive farm individuals with low morphological scores and eigenvalues from free‐roaming Singaporean junglefowl, validating our approach (Figure 3). Two exceptional free‐roaming individuals from Rifle Range clustered with farm individuals rather than with other free roamers (Figures 3 and 4). These individuals combined a domestic‐like genomic profile with low morphology scores, strongly suggesting they constitute domestic escapees and illustrating the potential for gene flow between domestics and free‐roaming red junglefowl in Singapore.

We removed captive farm individuals and verified that morphological parameters continue to predict the extent of domestic admixture in dataset 2, which does not contain historic samples, but not in dataset 3 (Figure S6). The lack of significance for this relationship in dataset 3 may be related to the substantially lower number of genome‐wide markers (7,812 SNPs in dataset 3 versus >4 million in dataset 2) and lower levels of linkage disequilibrium as per normal of ddRADSeq data (Peterson et al., 2012), leading to a lack of resolution.

### 4.3 Error rates of morphological diagnosis

Individuals with a morphology score greater than 75 invariably exhibited genomic profiles characteristic of wild‐type birds (Figure 3, right‐hand panels). There were zero false positives that would have combined a morphology score > 75 with a domestic‐like genotype. In the context of population management of Singapore's red junglefowl, we hence propose using a morphological threshold of 75 as a reasonably reliable indicator of birds with a genomic signature typical of wild red junglefowl in Singapore.

Conversely, the maximum morphology score for captive domestic farm individuals was 18.75, suggesting a conservative upper morphology cutoff of 25 to identify domestic chickens (Figure 3). Again, this value appeared fairly reliable, although the extended dataset (dataset 3), including ddRADSeq samples, does contain two false positives with low morphology scores (<25) that have genomic signatures representative of a number of fairly wild‐type individuals (Figure 3c, right‐hand panel, lower left corner).

Individuals with intermediate morphological scores, greater than 25 but less than 75, are likely to fall somewhere along the domestic introgression gradient. In datasets 1 and 2, which contained only whole‐genome resequenced individuals (*n* = 35 and *n* = 33, respectively), the majority of these morphologically intermediate birds (*n* = 17 in both datasets) displayed genomic signatures fairly similar to those of wild references and their proxies (Figure 3a, b). However, upon inclusion of additional individuals in the extended dataset (dataset 3) (*n* = 48), a number of birds with morphology scores between 25 and 75 (*n* = 27) exhibited a signature of more pronounced domestic contributions in their genomic profiles (Figure 3c, center of right‐hand panel). While the number of genetic markers in dataset 3 is much below that of datasets 1 and 2, these markers are—at the same time—at a much lower linkage disequilibrium (Peterson et al., 2012), reinforcing the need for a conservative cutoff at morphological score 75 as an indicator of wild‐type individuals.

### 4.4 The use of morphology in conservation assessments and decisions

The use of morphological characters in predicting genotypes has always been fraught with imprecision (Brisbin & Peterson, 2007; Condon, 2012; Daniels et al., 1998). This challenge is to be expected since morphological assessment is based on 1–2 dozen traits reflecting a few dozen genetic markers. When compared to the 702,902 genomic markers used in our linear regression, it is expected that assignments based on morphology scores are subject to stochastic imprecision.

In this context, it is encouraging to see a complete absence of false positives in our dataset when setting the lower morphological threshold for wild junglefowl at a score of 75. While we consider this cutoff reasonable for population managers, it does produce numerous false negatives (Figure 3), that is, individuals with morphological signs of introgression that belie their relatively wild‐type genomic profile. However, an argument can be made that—despite a genomic profile that would suggest otherwise—such false negatives are undesirable for population managers who are intent on removing individuals with obvious visible signs of domestic introgression. With our results, we recommend the removal of red junglefowl with morphology scores <75 to maintain the wild genotype in Singapore's recolonized population.

### 4.5 Morphological traits differ in their utility to predict genotype

Of the 17 traits considered, modeling analysis allowed us to identify a total of two (tarsus and primary feather coloration) and three traits (tail feather, primary feather, and lappet coloration) that best predict genomic profiles in females and males, respectively. Identifying such highly predictive traits is useful for providing population managers with criteria in a triage process that would see individuals which do not pass this first test subjected to further evaluation using additional traits.

The morphological traits identified as being of high utility in this study have the advantage of being scorable by anyone with a set of binoculars or camera. Although characters such as comb length, wattle length, and spur width have been used in previous studies to distinguish wild red junglefowl from domestics (Brisbin & Peterson, 2007; Condon, 2012; Nishida et al., 2000; Pheasantry & Pradesh, 2004), their major disadvantage when assessing wild populations is that they require the capture of the individual. Another commonly used morphological character is the presence of an eclipse plumage in males (Brisbin & Peterson, 2007; Condon, 2012; Nishida et al., 2000; Peterson & Brisbin, 1998; Pheasantry & Pradesh, 2004; Subhani, Awan, & Anwar, 2010). However, this feature is visible only during the breeding season and is not useful all year round.

Here, we present evidence for the reliability of the identified discrete morphological traits in predicting the extent of domestic introgression, without passing judgment on the suitability of continuous traits for the same purpose. Combining the traits identified by us with continuous traits found to reflect genomic profile in previous studies (Condon, 2012; Peterson & Brisbin, 2005) may further improve this prediction process.

### 4.6 Conservation

Species conservation in the presence of introgression has been a contentious topic (vonHoldt, Brzeski, Wilcove, & Rutledge, 2018). While some conservationists continue to debate whether the natural process of admixture and introgression can be viewed through a positive lens even when humans cause it in nature, many other conservation practitioners have made up their mind to try to counteract human‐caused introgression in wild animals and plants (Chattopadhyay, Garg, Mendenhall, et al., 2019; Chattopadhyay, Garg, Soo, et al., 2019; Nash et al., 2018). Our study helps conservation managers in a recolonized portion of the native range of the red junglefowl reduce the effects of continual introgression between domestic or feralized chickens and their wild counterparts.

Some researchers have gone so far as to question the continued existence of “pure” red junglefowl as a wild species, given thousands of years of potential contact with domesticated village chickens (Gering et al., 2015; Peterson & Brisbin, 1998). On the other hand, throughout South‐East Asia—even in urban settings within megalopolis cities—red junglefowl with a virtually perfect wild‐type morphology continue to persist. In this study, we detected a morphological and genomic continuum of free‐roaming junglefowl in Singapore, supporting the view that many wild populations in the species’ native range are likely affected by domestic introgression to some degree. This finding changes the conservation perspective regarding wild *Gallus gallus*, undoubtedly one of the most important bird species on Earth, whose status assessment has hitherto been guided by considerations of habitat loss and hunting, but not domestic introgression (BirdLife International, 2019).

Barring the availability of ancient DNA from paleontological deposits, it is a moot point to speculate to what extent contemporary wild junglefowl genomically resemble those from predomestication times. Incorporating two historic reference samples, we were able to make predictions regarding the reliability of morphological traits with a zero false‐positive rate. Many free‐roaming populations of red junglefowl across the native species range continue to exhibit a discrete and uniform set of wild‐type morphological traits, to which at least some of Singapore's birds also conform. Similar studies in other native populations—perhaps those that are in equilibrium and have never undergone extinction and recolonization—would be helpful in determining whether our findings can be applied more widely among wild junglefowl. In addition, data from other localities, and from museum specimens in particular, will be invaluable in determining whether our criteria for identifying admixed individuals can be applied to other populations as well. Meanwhile, the insights produced by this study provide a blueprint for conservation managers to identify birds likely to exhibit wild‐type genomic profiles. Our research shows that even junglefowl populations in relatively urban Asian regions can contribute to the global conservation and safeguarding of the wild allelic diversity in this species when properly managed.

## CONFLICT OF INTEREST

None delcared.

## Supporting information

Supplementary Material

## Data Availability

The data that support the findings of this study are openly available in the Sequence Read Archive under BioProject PRJNA629908. Additionally, photographic data that support the findings of this study are available in the Supporting information of this article.
