# Supplementary material for: Historic and modern genomes unveil a domestic introgression gradient in a wild red junglefowl population
Source: Evol Appl. 2020 Jun 4;13(9):2300–15. doi: 10.1111/eva.13023 (PMC7513718; doi:10.1111/eva.13023)
Supplement: Supplementary file 1 — Supplementary Material [file EVA-13-2300-s001.docx]

**SUPPLEMENTARY TABLE S1** Sample information of museum specimens.

ZRC: Zoological Reference Collection of the Lee Kong Chian Natural History Museum at the National University of Singapore; BMNH: British Museum of Natural History (Tring).

| **Voucher number and holding institution** | **Collection date** | **Locality** |
| --- | --- | --- |
| ZRC.3.1664 | 30 Jan 1911 | Temerloh, Pahang, Malay Peninsula |
| BMNH 1880.1.1.3247 | “before 1880” | Malacca, Malay Peninsula |

**SUPPLEMENTARY TABLE S2** List of whole genome resequenced datasets available on Sequence Read Archive or European Nucleotide Archive for *Gallus gallus* and used in this study.

| **Sample/Experiment Accession Number** | **Sample Name** | **Source** |
| --- | --- | --- |
| SAMEA5160201 | Red Junglefowl in Thailand | Qanbari *et al.*, 2019 |
| SAMEA5160202 | Red Junglefowl in Thailand | Qanbari *et al.*, 2019 |
| SAMEA5160203 | Red Junglefowl in Thailand | Qanbari *et al.*, 2019 |
| SAMEA5160204 | Red Junglefowl in Thailand | Qanbari *et al.*, 2019 |
| SAMEA5160205 | Red Junglefowl in Thailand | Qanbari *et al.*, 2019 |
| SAMEA5160206 | Red Junglefowl in Thailand | Qanbari *et al.*, 2019 |
| SAMEA5160207 | Red Junglefowl in Thailand | Qanbari *et al.*, 2019 |
| SAMEA5160208 | Red Junglefowl in Thailand | Qanbari *et al.*, 2019 |
| SAMEA5160209 | Red Junglefowl in Thailand | Qanbari *et al.*, 2019 |
| SAMEA5160210 | Red Junglefowl in Thailand | Qanbari *et al.*, 2019 |
| SAMEA5160211 | Red Junglefowl in Thailand | Qanbari *et al.*, 2019 |
| SAMEA5160212 | Red Junglefowl in Thailand | Qanbari *et al.*, 2019 |
| SAMEA5160213 | Red Junglefowl in Thailand | Qanbari *et al.*, 2019 |
| SAMEA5160214 | Red Junglefowl in Thailand | Qanbari *et al.*, 2019 |
| SAMEA5160215 | Red Junglefowl in Thailand | Qanbari *et al.*, 2019 |
| SAMEA5160216 | Red Junglefowl in Thailand | Qanbari *et al.*, 2019 |
| SAMEA5160217 | Red Junglefowl in Thailand | Qanbari *et al.*, 2019 |
| SAMEA5160218 | Red Junglefowl in Thailand | Qanbari *et al.*, 2019 |
| SAMEA5160219 | Red Junglefowl in Thailand | Qanbari *et al.*, 2019 |
| SAMEA5160220 | Red Junglefowl in Thailand | Qanbari *et al.*, 2019 |
| SAMEA5160221 | Red Junglefowl in Thailand | Qanbari *et al.*, 2019 |
| SAMEA5160222 | Red Junglefowl in Thailand | Qanbari *et al.*, 2019 |
| SAMEA5160223 | Red Junglefowl in Thailand | Qanbari *et al.*, 2019 |
| SAMEA5160224 | Red Junglefowl in Thailand | Qanbari *et al.*, 2019 |
| SAMEA5160225 | Red Junglefowl in India | Qanbari *et al.*, 2019 |
| SAMEA5160226 | Red Junglefowl in India | Qanbari *et al.*, 2019 |
| SAMEA5160227 | Red Junglefowl in India | Qanbari *et al.*, 2019 |
| SAMEA5160228 | Red Junglefowl in India | Qanbari *et al.*, 2019 |
| SAMEA5160229 | Red Junglefowl in India | Qanbari *et al.*, 2019 |
| SAMEA5160230 | Red Junglefowl in India | Qanbari *et al.*, 2019 |
| SAMEA5160231 | Red Junglefowl in India | Qanbari *et al.*, 2019 |
| SAMEA5160232 | Red Junglefowl in India | Qanbari *et al.*, 2019 |
| SAMEA5160233 | Red Junglefowl in India | Qanbari *et al.*, 2019 |
| SAMEA5160234 | Red Junglefowl in India | Qanbari *et al.*, 2019 |
| SAMEA5160235 | Commercial Broiler Line A | Qanbari *et al.*, 2019 |
| SAMEA5160236 | Commercial Broiler Line A | Qanbari *et al.*, 2019 |
| SAMEA5160237 | Commercial Broiler Line A | Qanbari *et al.*, 2019 |
| SAMEA5160238 | Commercial Broiler Line A | Qanbari *et al.*, 2019 |
| SAMEA5160239 | Commercial Broiler Line A | Qanbari *et al.*, 2019 |
| SAMEA5160255 | Commercial Broiler Line B | Qanbari *et al.*, 2019 |
| SAMEA5160256 | Commercial Broiler Line B | Qanbari *et al.*, 2019 |
| SAMEA5160257 | Commercial Broiler Line B | Qanbari *et al.*, 2019 |
| SAMEA5160258 | Commercial Broiler Line B | Qanbari *et al.*, 2019 |
| SAMEA5160259 | Commercial Broiler Line B | Qanbari *et al.*, 2019 |
| SAMEA5160275 | Commercial Brown Layer | Qanbari *et al.*, 2019 |
| SAMEA5160276 | Commercial Brown Layer | Qanbari *et al.*, 2019 |
| SAMEA5160277 | Commercial Brown Layer | Qanbari *et al.*, 2019 |
| SAMEA5160278 | Commercial Brown Layer | Qanbari *et al.*, 2019 |
| SAMEA5160279 | Commercial Brown Layer | Qanbari *et al.*, 2019 |
| SAMEA5160300 | Commercial White Layer | Qanbari *et al.*, 2019 |
| SAMEA5160301 | Commercial White Layer | Qanbari *et al.*, 2019 |
| SAMEA5160302 | Commercial White Layer | Qanbari *et al.*, 2019 |
| SAMEA5160303 | Commercial White Layer | Qanbari *et al.*, 2019 |
| SAMEA5160304 | Commercial White Layer | Qanbari *et al.*, 2019 |
| SAMEA5160325 | Pooled Commercial Broiler Line D | Qanbari *et al.*, 2019 |
| SAMEA5160326 | Pooled Rhode Island White | Qanbari *et al.*, 2019 |
| SRX6715081 | Dulong Chicken | Sichuan Agriculture University |
| SRX6717795 | Dulong Chicken | Sichuan Agriculture University |
| SRX3993586 | DNAseq-of-Ethioian-Horro3-indigenous-chicken | Lawal *et al.*, 2018 |
| SRX3993585 | DNAseq-of-Ethioian-Horro4-indigenous-chicken | Lawal *et al.*, 2018 |
| SRX3993581 | DNAseq-of-Ethioian-Jarso2-indigenous-chicken | Lawal *et al.*, 2018 |
| SRX3993594 | DNAseq-of-Ethioian-Jarso5-indigenous-chicken | Lawal *et al.*, 2018 |
| SRX3993591 | DNAseq-of-Saudi-Arabian3-indigenous-chicken | Lawal *et al.*, 2018 |
| SRX3993597 | DNAseq-of-Saudi-Arabian5-indigenous-chicken | Lawal *et al.*, 2018 |
| SRX3993575 | DNAseq-of-Sri-Lankan2-indigenous-chicken | Lawal *et al.*, 2018 |
| SRX3993573 | DNAseq-of-Sri-Lankan4-indigenous-chicken | Lawal *et al.*, 2018 |
| SRX3299129 | DNA-seq of Gallus gallus: whole blood | Sohn *et al*., 2018 |
| SRX3299128 | DNA-seq of Gallus gallus: whole blood | Sohn *et al*., 2018 |
| DRX083736 | Illumina HiSeq 2500 paired end sequencing of SAMD00077894 | Ulfah *et al*., 2016 |
| DRX083735 | Illumina HiSeq 2500 paired end sequencing of SAMD00077893 | Ulfah *et al*., 2016 |
| DRX083727 | Illumina HiSeq 2500 paired end sequencing of SAMD00077885 | Ulfah *et al*., 2016 |
| DRX083726 | Illumina HiSeq 2500 paired end sequencing of SAMD00077884 | Ulfah *et al*., 2016 |
| DRX083717 | Illumina HiSeq 2500 paired end sequencing of SAMD00077875 | Ulfah *et al*., 2016 |
| DRX083715 | Illumina HiSeq 2500 paired end sequencing of SAMD00077873 | Ulfah *et al*., 2016 |
| DRX083708 | Illumina HiSeq 2500 paired end sequencing of SAMD00077866 | Ulfah *et al*., 2016 |
| DRX083707 | Illumina HiSeq 2500 paired end sequencing of SAMD00077865 | Ulfah *et al*., 2016 |
| DRX083703 | Illumina HiSeq 2500 paired end sequencing of SAMD00077861 | Ulfah *et al*., 2016 |
| DRX083702 | Illumina HiSeq 2500 paired end sequencing of SAMD00077860 | Ulfah *et al*., 2016 |
| SRX2578635 | - | He *et al.*, 2017 |
| SRX2578634 | - | He *et al.*, 2017 |
| SRX1561301 | Genome Resequencing of Tibetan chicken : blood from sample WL-E | Zhang *et al.*, 2016 |
| SRX1561295 | Genome Resequencing of Tibetan chicken : blood from sample LD | Zhang *et al.*, 2016 |
| SRX1121841 | Araucana Genome | Oh *et al.*, 2016 |
| SRX1121840 | Araucana Genome | Oh *et al.*, 2016 |
| SRX1121838 | Korean Domestic Chicken Genome | Oh *et al.*, 2016 |
| SRX1121837 | Korean Domestic Chicken Genome | Oh *et al.*, 2016 |
| SRX1121835 | white leghorn genome | Oh *et al.*, 2016 |
| SRX1121834 | white leghorn genome | Oh *et al.*, 2016 |
| SRX511225 | Dometication and adaption of Tibetan chicken | Wang *et al.*, 2015 |
| SRX511221 | Dometication and adaption of Tibetan chicken | Wang *et al.*, 2015 |
| SRX511212 | Dometication and adaption of Tibetan chicken | Wang *et al.*, 2015 |
| SRX511208 | Dometication and adaption of Tibetan chicken | Wang *et al.*, 2015 |
| SRX511201 | Dometication and adaption of Tibetan chicken | Wang *et al.*, 2015 |
| SRX511200 | Dometication and adaption of Tibetan chicken | Wang *et al.*, 2015 |
| SRX286799 | Taiwanese native chicken L2 genomic reads | Fan *et al.*, 2013 |
| SRX286798 | Taiwanese native chicken L2 genomic reads | Fan *et al.*, 2013 |
| SRX286776 | Silkie chicken genome sequencing | Fan *et al.*, 2013 |
| SRX286765 | Silkie chicken genome sequencing | Fan *et al.*, 2013 |

**SUPPLEMENTARY TABLE S3** List of fitted linear models based on the subset of morphological traits displaying an above-average contribution to a domestic-wild morphology cline. The most important traits identified in males are coloration of hackles, tail, primaries, lesser coverts, greater and secondary coverts, rump plumes and lappet. The most important traits identified in females are the coloration of hackles, tail, primaries, lesser coverts, greater and secondary coverts and tarsus.

|  | **Model** | **AIC** |
| --- | --- | --- |
| Males | Eigenvalues~Hack+Tail+Pri+LC+GSC+RP+Lap | -20.671956 |
|  | Eigenvalues~Hack+Tail+Pri+LC+GSC+RP | -18.77202 |
|  | Eigenvalues~Hack+Tail+Pri+LC+GSC+Lap | -22.371722 |
|  | Eigenvalues~Hack+Tail+Pri+LC+RP+Lap | -22.449439 |
|  | Eigenvalues~Hack+Tail+Pri+GSC+RP+Lap | -22.317184 |
|  | Eigenvalues~Hack+Tail+LC+GSC+RP+Lap | -9.741487 |
|  | Eigenvalues~Hack+Pri+LC+GSC+RP+Lap | -18.730811 |
|  | Eigenvalues~Tail+Pri+LC+GSC+RP+Lap | -22.138021 |
|  | Eigenvalues~Pri | -19.712138 |
|  | Eigenvalues~Hack | -9.034895 |
|  | Eigenvalues~Tail | -9.88997 |
|  | Eigenvalues~LC | -11.555033 |
|  | Eigenvalues~GSC | -9.716831 |
|  | Eigenvalues~RP | -10.358063 |
|  | Eigenvalues~Lap | -9.009032 |
|  | Eigenvalues~Hack+LC+GSC+RP+Lap | -10.120163 |
|  | Eigenvalues~Hack+Pri+GSC+RP+Lap | -20.67985 |
|  | Eigenvalues~Hack+Pri+LC+GSC+Lap | -18.108134 |
|  | Eigenvalues~Hack+Pri+LC+GSC+RP | -18.81931 |
|  | Eigenvalues~Hack+Pri+LC+RP+Lap | -20.383205 |
|  | Eigenvalues~Hack+Tail+GSC+RP+Lap | -9.296204 |
|  | Eigenvalues~Hack+Tail+LC+GSC+Lap | -11.737288 |
|  | Eigenvalues~Hack+Tail+LC+GSC+RP | -11.146086 |
|  | Eigenvalues~Hack+Tail+LC+RP+Lap | -11.542576 |
|  | Eigenvalues~Hack+Tail+Pri+GSC+Lap | -24.161858 |
|  | Eigenvalues~Hack+Tail+Pri+GSC+RP | -20.57623 |
|  | Eigenvalues~Hack+Tail+Pri+LC+GSC | -18.78028 |
|  | Eigenvalues~Hack+Tail+Pri+LC+Lap | -24.25597 |
|  | Eigenvalues~Hack+Tail+Pri+LC+RP | -18.904074 |
|  | Eigenvalues~Hack+Tail+Pri+RP+Lap | -24.242409 |
|  | Eigenvalues~Pri+LC+GSC+RP+Lap | -20.325506 |
|  | Eigenvalues~Tail+LC+GSC+RP+Lap | -9.0361 |
|  | Eigenvalues~Tail+Pri+GSC+RP+Lap | -24.063992 |
|  | Eigenvalues~Tail+Pri+LC+GSC+Lap | -23.998414 |
|  | Eigenvalues~Tail+Pri+LC+GSC+RP | -20.701783 |
|  | Eigenvalues~Tail+Pri+LC+RP+Lap | -24.002886 |
|  | Eigenvalues~Hack+GSC+RP+Lap | -9.194042 |
|  | Eigenvalues~Hack+LC+GSC+Lap | -11.445894 |
|  | Eigenvalues~Hack+LC+GSC+RP | -11.930188 |
|  | Eigenvalues~Hack+LC+RP+Lap | -11.831337 |
|  | Eigenvalues~Hack+Pri+GSC+Lap | -19.85187 |
|  | Eigenvalues~Hack+Pri+GSC+RP | -20.776705 |
|  | Eigenvalues~Hack+Pri+LC+GSC | -16.416082 |
|  | Eigenvalues~Hack+Pri+LC+Lap | -20.078638 |
|  | Eigenvalues~Hack+Pri+LC+RP | -19.243051 |
|  | Eigenvalues~Hack+Pri+RP+Lap | -22.383205 |
|  | Eigenvalues~Hack+Tail+GSC+Lap | -11.061687 |
|  | Eigenvalues~Hack+Tail+GSC+RP | -10.967459 |
|  | Eigenvalues~Hack+Tail+LC+GSC | -12.911151 |
|  | Eigenvalues~Hack+Tail+LC+Lap | -13.539677 |
|  | Eigenvalues~Hack+Tail+LC+RP | -12.390613 |
|  | Eigenvalues~Hack+Tail+Pri+GSC | -20.778522 |
|  | Eigenvalues~Hack+Tail+Pri+Lap | -26.118934 |
|  | Eigenvalues~Hack+Tail+Pri+LC | -19.338846 |
|  | Eigenvalues~Hack+Tail+Pri+RP | -20.877694 |
|  | Eigenvalues~Hack+Tail+RP+Lap | -9.744747 |
|  | Eigenvalues~LC+GSC+RP+Lap | -9.726791 |
|  | Eigenvalues~Pri+GSC+RP+Lap | -22.315636 |
|  | Eigenvalues~Pri+LC+GSC+Lap | -20.08728 |
|  | Eigenvalues~Pri+LC+GSC+RP | -20.717365 |
|  | Eigenvalues~Pri+LC+RP+Lap | -22.075817 |
|  | Eigenvalues~Tail+GSC+RP+Lap | -9.640862 |
|  | Eigenvalues~Tail+LC+GSC+Lap | -10.841993 |
|  | Eigenvalues~Tail+LC+GSC+RP | -10.279325 |
|  | Eigenvalues~Tail+LC+RP+Lap | -10.465357 |
|  | Eigenvalues~Tail+Pri+GSC+Lap | -25.956527 |
|  | Eigenvalues~Tail+Pri+GSC+RP | -22.573021 |
|  | Eigenvalues~Tail+Pri+LC+GSC | -20.688297 |
|  | Eigenvalues~Tail+Pri+LC+Lap | -25.915366 |
|  | Eigenvalues~Tail+Pri+LC+RP | -20.891991 |
|  | Eigenvalues~Tail+Pri+RP+Lap | -25.978275 |
|  | Eigenvalues~GSC+RP+Lap | -9.905777 |
|  | Eigenvalues~Hack+GSC+Lap | -8.759042 |
|  | Eigenvalues~Hack+GSC+RP | -11.170243 |
|  | Eigenvalues~Hack+LC+GSC | -12.752195 |
|  | Eigenvalues~Hack+LC+Lap | -13.334522 |
|  | Eigenvalues~Hack+LC+RP | -13.278553 |
|  | Eigenvalues~Hack+Pri+GSC | -17.749992 |
|  | Eigenvalues~Hack+Pri+Lap | -21.731877 |
|  | Eigenvalues~Hack+Pri+LC | -17.556926 |
|  | Eigenvalues~Hack+Pri+RP | -21.11302 |
|  | Eigenvalues~Hack+RP+Lap | -9.071654 |
|  | Eigenvalues~Hack+Tail+GSC | -12.263864 |
|  | Eigenvalues~Hack+Tail+Lap | -11.669505 |
|  | Eigenvalues~Hack+Tail+LC | -14.211031 |
|  | Eigenvalues~Hack+Tail+Pri | -20.982886 |
|  | Eigenvalues~Hack+Tail+RP | -10.247217 |
|  | Eigenvalues~LC+GSC+Lap | -10.341015 |
|  | Eigenvalues~LC+GSC+RP | -11.400869 |
|  | Eigenvalues~LC+RP+Lap | -11.046293 |
|  | Eigenvalues~Pri+GSC+Lap | -21.698241 |
|  | Eigenvalues~Pri+GSC+RP | -22.7145 |
|  | Eigenvalues~Pri+LC+GSC | -18.087459 |
|  | Eigenvalues~Pri+LC+Lap | -22.061253 |
|  | Eigenvalues~Pri+LC+RP | -21.24229 |
|  | Eigenvalues~Pri+RP+Lap | -23.986792 |
|  | Eigenvalues~Tail+GSC+Lap | -11.136446 |
|  | Eigenvalues~Tail+GSC+RP | -11.147439 |
|  | Eigenvalues~Tail+LC+GSC | -11.264257 |
|  | Eigenvalues~Tail+LC+Lap | -12.390538 |
|  | Eigenvalues~Tail+LC+RP | -10.59804 |
|  | Eigenvalues~Tail+Pri+GSC | -22.680144 |
|  | Eigenvalues~Tail+Pri+Lap | -27.900529 |
|  | Eigenvalues~Tail+Pri+LC | -20.960095 |
|  | Eigenvalues~Tail+Pri+RP | -22.87567 |
|  | Eigenvalues~Tail+RP+Lap | -9.797111 |
|  | Eigenvalues~Hack+GSC | -10.134182 |
|  | Eigenvalues~Hack+Lap | -9.092433 |
|  | Eigenvalues~Hack+Tail | -11.455115 |
|  | Eigenvalues~Hack+Pri | -17.873214 |
|  | Eigenvalues~Hack+LC | -14.269079 |
|  | Eigenvalues~Hack+RP | -10.388719 |
|  | Eigenvalues~Tail+Lap | -11.546999 |
|  | Eigenvalues~Tail+Pri | -22.794143 |
|  | Eigenvalues~Tail+LC | -11.589614 |
|  | Eigenvalues~Tail+GSC | -11.708727 |
|  | Eigenvalues~Tail+RP | -9.685619 |
|  | Eigenvalues~Pri+RP | -23.091461 |
|  | Eigenvalues~Pri+LC | -18.896853 |
|  | Eigenvalues~Pri+GSC | -19.674398 |
|  | Eigenvalues~Pri+Lap | -23.559834 |
|  | Eigenvalues~LC+GSC | -10.911141 |
|  | Eigenvalues~LC+RP | -11.898898 |
|  | Eigenvalues~LC+Lap | -12.019159 |
|  | Eigenvalues~GSC+Lap | -8.915169 |
|  | Eigenvalues~GSC+RP | -11.807672 |
|  | Eigenvalues~Lap+RP | -9.520071 |
| Females | Eigenvalues~Hack+Tail+LC+Pri+GSC+TarC | -16.864967 |
|  | Eigenvalues~Hack+Tail+LC+Pri+GSC | -12.886316 |
|  | Eigenvalues~Hack+Tail+LC+Pri+TarC | -18.729888 |
|  | Eigenvalues~Hack+Tail+LC+GSC+TarC | -14.69145 |
|  | Eigenvalues~Hack+Tail+Pri+GSC+TarC | -17.97226 |
|  | Eigenvalues~Tail+LC+Pri+GSC+TarC | -16.864967 |
|  | Eigenvalues~Hack | -15.307358 |
|  | Eigenvalues~Tail | -15.307358 |
|  | Eigenvalues~LC | -13.350019 |
|  | Eigenvalues~Pri | -15.918793 |
|  | Eigenvalues~GSC | -8.390916 |
|  | Eigenvalues~TarC | -14.079291 |
|  | Eigenvalues~Hack+LC+GSC+TarC | -14.69145 |
|  | Eigenvalues~Hack+LC+Pri+GSC | -12.886316 |
|  | Eigenvalues~Hack+LC+Pri+TarC | -18.729888 |
|  | Eigenvalues~Hack+Pri+GSC+TarC | -17.97226 |
|  | Eigenvalues~Hack+Tail+GSC+TarC | -13.573409 |
|  | Eigenvalues~Hack+Tail+LC+GSC | -13.266901 |
|  | Eigenvalues~Hack+Tail+LC+Pri | -14.731691 |
|  | Eigenvalues~Hack+Tail+LC+TarC | -16.680611 |
|  | Eigenvalues~Hack+Tail+Pri+GSC | -14.336025 |
|  | Eigenvalues~Hack+Tail+Pri+TarC | -19.480188 |
|  | Eigenvalues~LC+Pri+GSC+TarC | -18.583511 |
|  | Eigenvalues~Tail+LC+GSC+TarC | -14.69145 |
|  | Eigenvalues~Tail+LC+Pri+GSC | -12.886316 |
|  | Eigenvalues~Tail+LC+Pri+TarC | -18.729888 |
|  | Eigenvalues~Tail+Pri+GSC+TarC | -17.97226 |
|  | Eigenvalues~Hack+GSC+TarC | -13.573409 |
|  | Eigenvalues~Hack+LC+GSC | -13.266901 |
|  | Eigenvalues~Hack+LC+Pri | -14.731691 |
|  | Eigenvalues~Hack+LC+TarC | -16.680611 |
|  | Eigenvalues~Hack+Pri+GSC | -14.336025 |
|  | Eigenvalues~Hack+Pri+TarC | -19.480188 |
|  | Eigenvalues~Hack+Tail+GSC | -13.321521 |
|  | Eigenvalues~Hack+Tail+LC | -14.932836 |
|  | Eigenvalues~Hack+Tail+Pri | -16.31782 |
|  | Eigenvalues~Hack+Tail+TarC | -15.434598 |
|  | Eigenvalues~LC+GSC+TarC | -16.423199 |
|  | Eigenvalues~LC+Pri+GSC | -13.052836 |
|  | Eigenvalues~LC+Pri+TarC | -20.563989 |
|  | Eigenvalues~Pri+GSC+TarC | -19.811125 |
|  | Eigenvalues~Tail+GSC+TarC | -13.573409 |
|  | Eigenvalues~Tail+LC+GSC | -13.266901 |
|  | Eigenvalues~Tail+LC+Pri | -14.731691 |
|  | Eigenvalues~Tail+LC+TarC | -16.680611 |
|  | Eigenvalues~Tail+Pri+GSC | -14.336025 |
|  | Eigenvalues~Tail+Pri+TarC | -19.480188 |
|  | Eigenvalues~Hack+Tail | -15.307358 |
|  | Eigenvalues~Hack+LC | -14.932836 |
|  | Eigenvalues~Hack+Pri | -16.31782 |
|  | Eigenvalues~Hack+GSC | -13.321521 |
|  | Eigenvalues~Hack+TarC | -15.434598 |
|  | Eigenvalues~Tail+LC | -14.932836 |
|  | Eigenvalues~Tail+Pri | -16.31782 |
|  | Eigenvalues~Tail+GSC | -13.321521 |
|  | Eigenvalues~Tail+TarC | -15.434598 |
|  | Eigenvalues~LC+Pri | -15.04241 |
|  | Eigenvalues~LC+GSC | -11.357683 |
|  | Eigenvalues~LC+TarC | -18.409394 |
|  | Eigenvalues~GSC+Pri | -14.22551 |
|  | Eigenvalues~TarC+Pri | -21.479253 |
|  | Eigenvalues~TarC+GSC | -13.917037 |

**
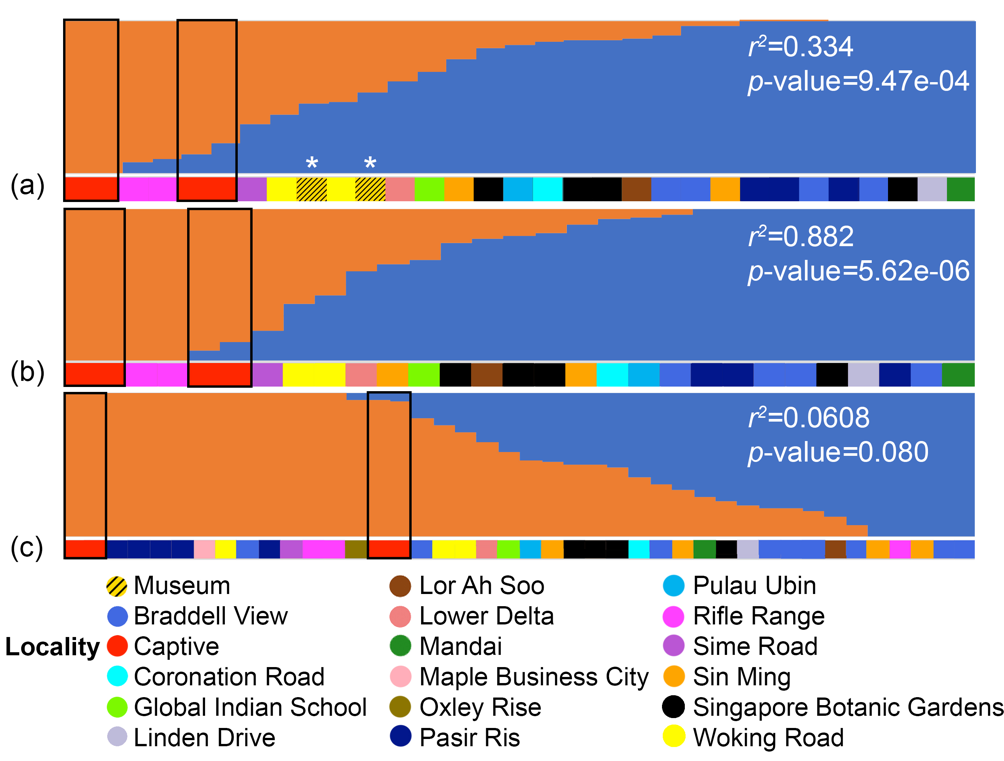
FIGURE S1** STRUCTURE plots: (a) whole-genome re-sequenced individuals including museum samples at *K* = 2 after removal of kin (n = 31) based on 100,000 single nucleotide polymorphisms; (b) modern whole-genome re-sequenced individuals excluding museum samples after removal of kin (n = 29) based on 100,000 single nucleotide polymorphisms; (c) all modern individuals excluding museum samples but including individuals from restriction-associated DNA sequencing after removal of kin (n = 42) based on 9,181 single nucleotide polymorphisms. Coefficients of determination and *p*-values from regression analysis of morphology scores against *q* scores are given in each STRUCTURE plot.


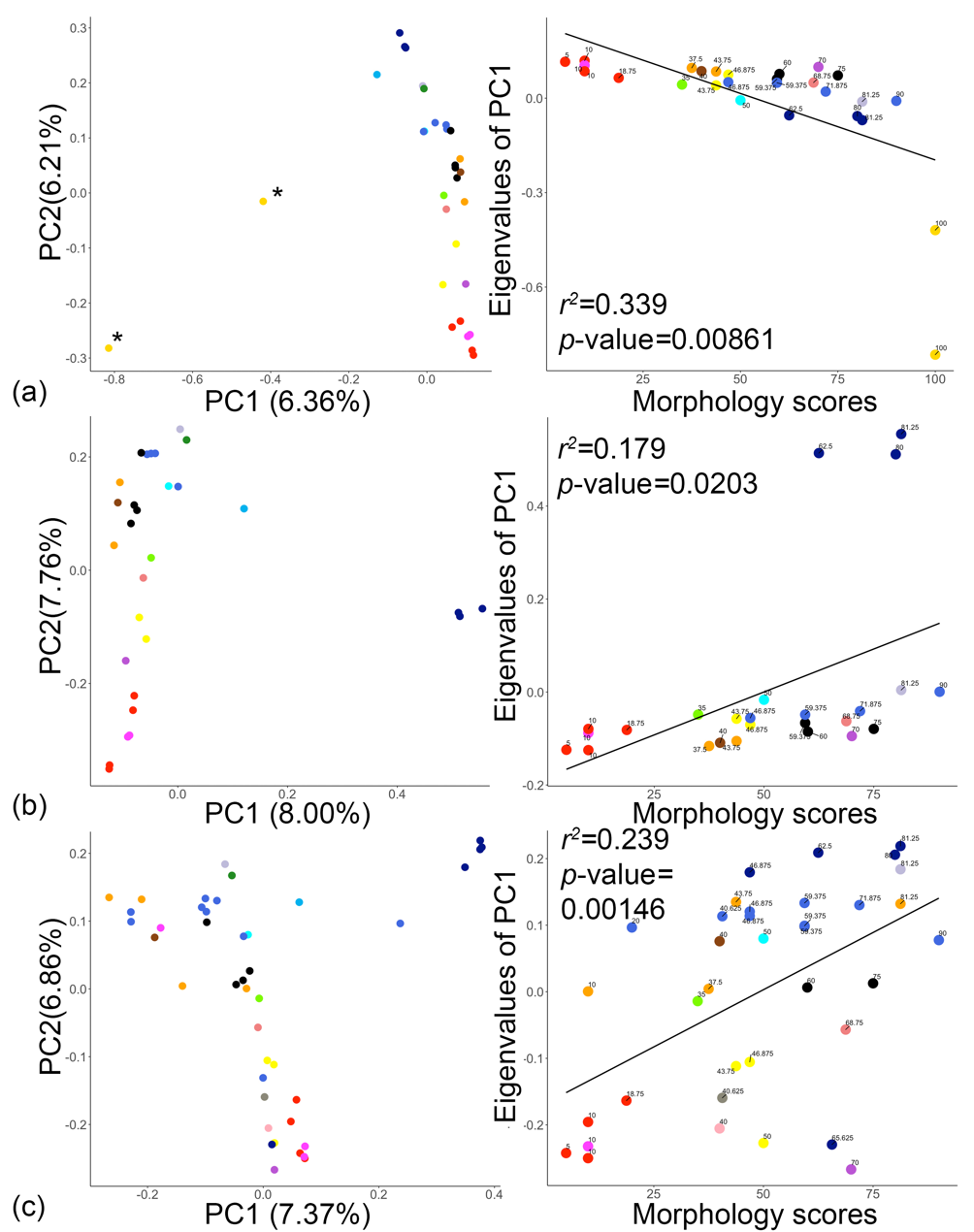


**FIGURE S2** Principal component analysis (left) and linear regression of morphological scores against eigenvalues extracted from PC1 (right): (a) all whole-genome re-sequenced individuals including museum samples after kin removal (n = 31), incorporating 10,079,620 single nucleotide polymorphisms; (b) modern whole-genome re-sequenced individuals excluding museum samples after kin removal (n = 29), incorporating 5,225,659 single nucleotide polymorphisms; (c) all modern individuals excluding museum samples but including individuals from restriction-associated DNA sequencing after kin removal (n = 42), incorporating 9,181 single nucleotide polymorphisms. The percentage of total variation explained by each principal component is shown in brackets. Coefficients of determination and *p*-values are reported for each plot. Colors refer to localities as shown in Figure S1.

**
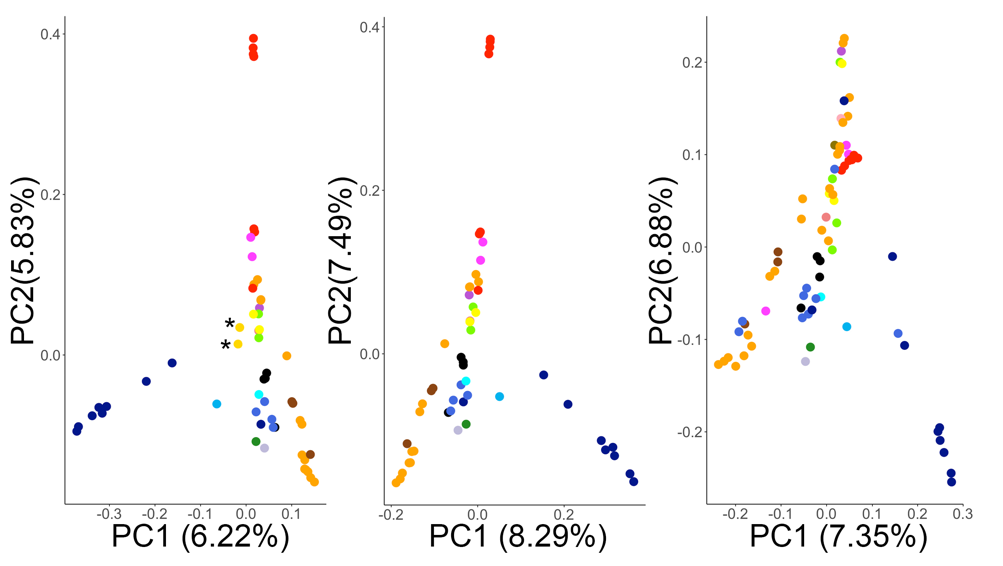
 FIGURE S3** Principal component analysis incorporating 10,134,987; 5,273,631; and 10,940 single nucleotide polymorphisms for datasets (1), (2), and (3), respectively (left to right). The percentage of total variation explained by each principal component is shown in brackets. Plot trifurcations emerging in all three datasets, with Pasir Ris, Sin Ming and captive individuals scattered on the three branches, are likely an artifact due to oversampling of certain localities. Colors refer to localities as shown in Figure S1.


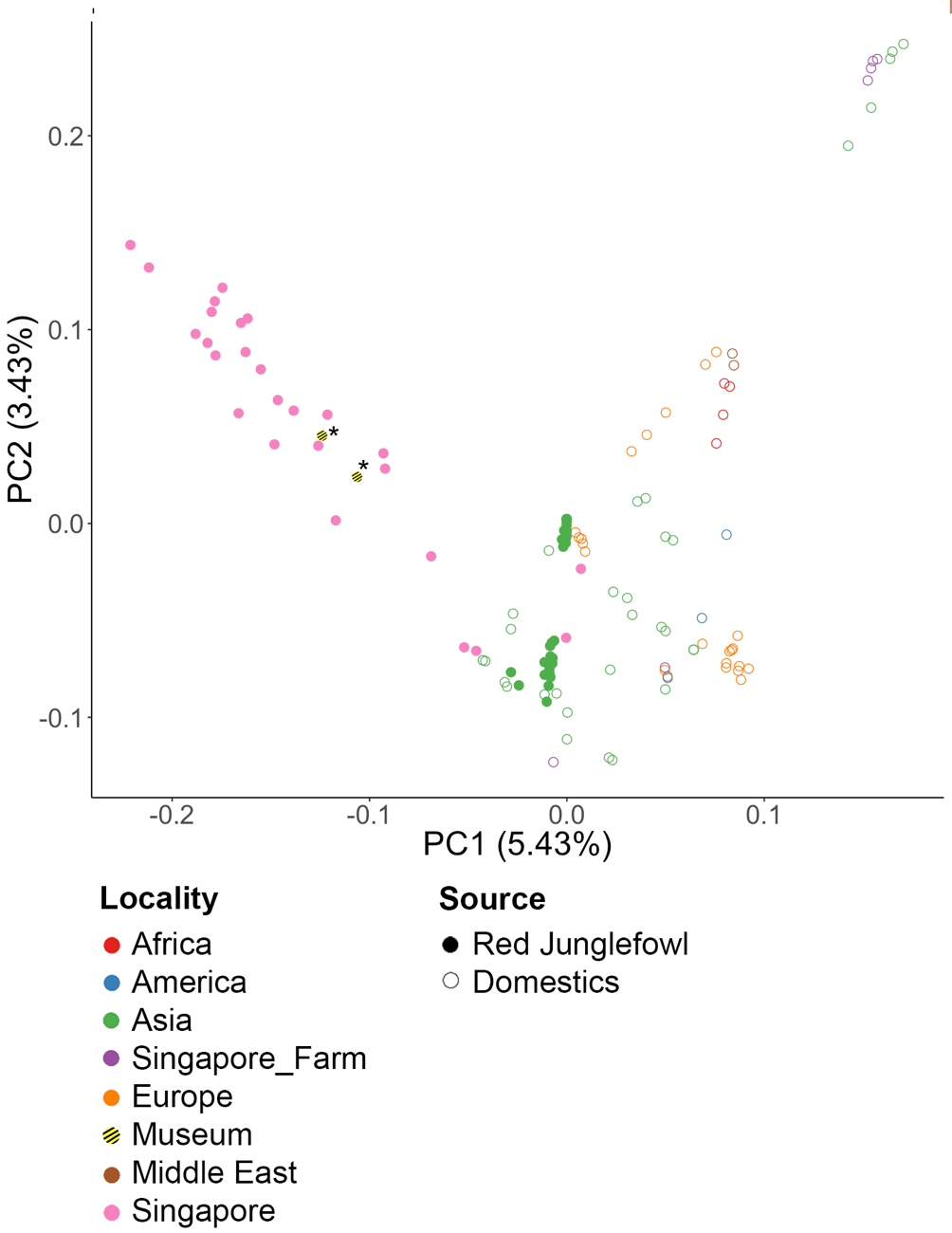


**FIGURE S4** Principal component analysis of the genomic dataset for individuals from Singapore and two historic Malaysian samples in relation to various other Red Junglefowls and chicken breeds based on 2,323,047 single nucleotide polymorphisms. The percentage of total variation explained by each principal component is shown in brackets. Museum samples are labeled with an asterisk (*).


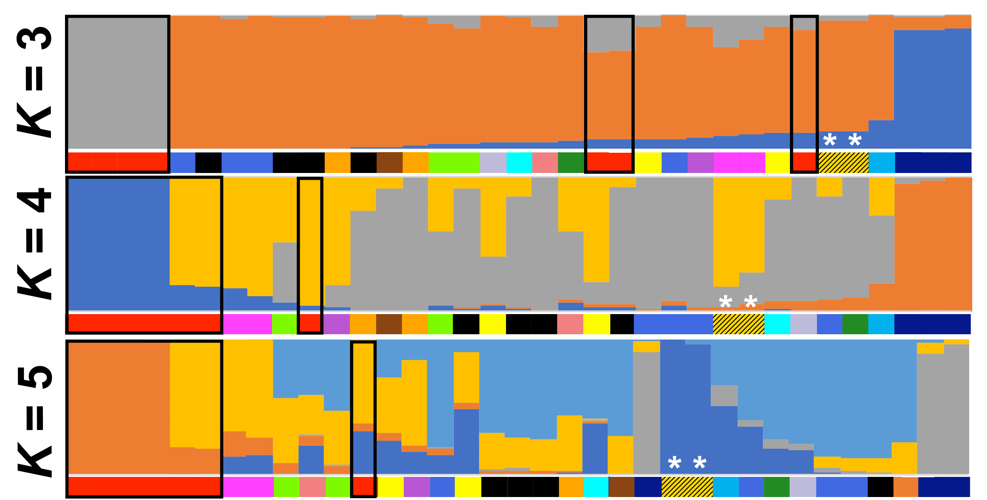


**FIGURE S5** STRUCTURE plots of whole-genome re-sequenced individuals including museum samples at *K* = 3 to 5 (n = 35) based on 100,000 single nucleotide polymorphisms. Colors refer to localities as shown in Figure S1.


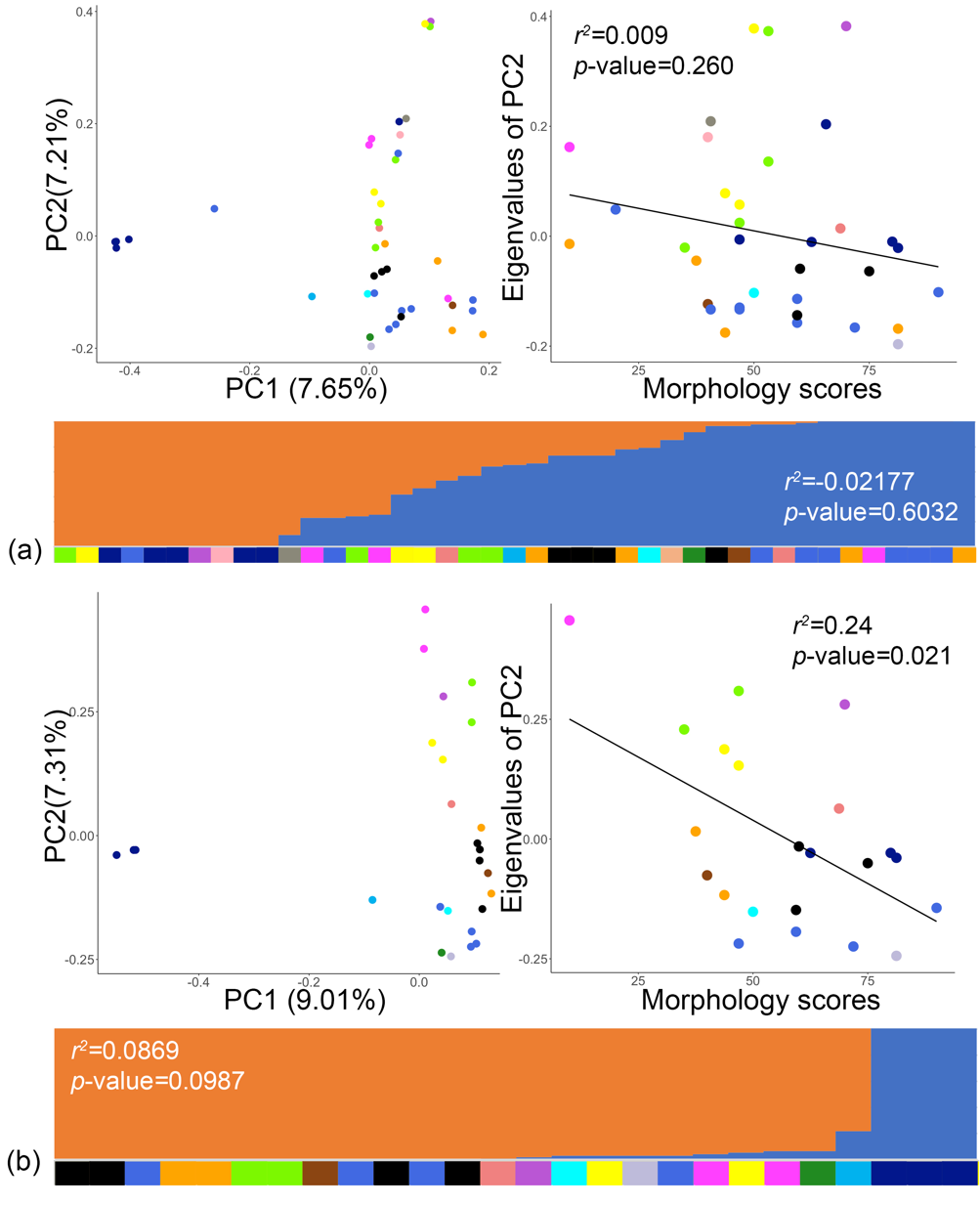


**FIGURE S6** Principal component analysis (upper left), linear regression of morphological scores against eigenvalues extracted from PC2 (upper right) and STRUCTURE plot at *K* = 2 (below): (a) all modern individuals excluding museum and captive samples but including individuals from restriction-associated DNA sequencing (dataset 3 minus captives; n = 41), incorporating 8,857 single nucleotide polymorphisms; (b) modern whole-genome re-sequenced individuals excluding museum and captive samples (dataset 2 minus captives; n = 26), incorporating 100,000 single nucleotide polymorphisms. Colors refer to localities as shown in Figure S1.

**Literature Cited**

Fan, W. L., Ng, C. S., Chen, C. F., Lu, M. Y. J., Chen, Y. H., Liu, C. J., ... & Lai, Y. T. (2013). Genome-wide patterns of genetic variation in two domestic chickens. *Genome Biology and Evolution*, 5(7), 1376-1392.

He, C., Chen, Y., Yang, K., Zhai, Z., Zhao, W., Liu, S., ... & Zhou, Z. (2017). Genetic pattern and gene localization of polydactyly in Beijing fatty chicken. *PloS ONE*, 12(5).

Lawal, R. A., Al-Atiyat, R. M., Aljumaah, R. S., Silva, P., Mwacharo, J. M., & Hanotte, O. (2018). Whole-genome resequencing of red junglefowl and indigenous village chicken reveal new insights on the genome dynamics of the species*. Frontiers in Genetics*, 9, 264.

Oh, D., Son, B., Mun, S., Oh, M. H., Oh, S., Ha, J., ... & Han, K. (2016). Whole genome re-sequencing of three domesticated chicken breeds. *Zoological Science*, 33(1), 73-77.

Qanbari, S., Rubin, C. J., Maqbool, K., Weigend, S., Weigend, A., Geibel, J., ... & Preisinger, R. (2019). Genetics of adaptation in modern chicken. *PLoS Genetics,* 15(4), e1007989.

Sohn, J. I., Nam, K., Hong, H., Kim, J. M., Lim, D., Lee, K. T., ... & Nam, J. W. (2018). Whole genome and transcriptome maps of the entirely black native Korean chicken breed *Yeonsan Ogye*. *GigaScience*, 7(7), giy086.

Ulfah, M., Kawahara-Miki, R., Farajalllah, A., Muladno, M., Dorshorst, B., Martin, A., & Kono, T. (2016). Genetic features of red and green junglefowls and relationship with Indonesian native chickens Sumatera and Kedu Hitam. *BMC Genomics*, 17(1), 320.

Wang, M. S., Li, Y., Peng, M. S., Zhong, L. I., Wang, Z. J., Li, Q. Y., ... & Yang, M. M. (2015). Genomic analyses reveal potential independent adaptation to high altitude in Tibetan chickens. *Molecular Biology and Evolution*, 32(7), 1880-1889.

Zhang, Q., Gou, W., Wang, X., Zhang, Y., Ma, J., Zhang, H., ... & Zhang, H. (2016). Genome resequencing identifies unique adaptations of Tibetan chickens to hypoxia and high-dose ultraviolet radiation in high-altitude environments. *Genome Biology and Evolution*, 8(3), 765-776.
